# Anticancer Activity of Encapsulated Pearl Millet Polyphenol-Rich Extract against Proliferating and Non-Proliferating Breast Cancer Cells In Vitro

**DOI:** 10.3390/cancers16091750

**Published:** 2024-04-30

**Authors:** Latifa Hajri, Anna Lewińska, Iwona Rzeszutek, Bernadetta Oklejewicz, Renata Wojnarowska-Nowak, Agnieszka Krogul-Sobczak, Ewa Szpyrka, Alfredo Aires, Soumaya Ghodbane, Mohamed Ammari, Maciej Wnuk

**Affiliations:** 1 Faculty of Sciences of Bizerte, Laboratory of Integrative Physiology, University of Carthage, Jarzouna, Bizerte 7021, Tunisia; hajrilatifa145@gmail.com (L.H.); ghodhbanes@yahoo.fr (S.G.); mohamed.ammari@fsb.rnu.tn (M.A.); 2 Institute of Biotechnology, College of Natural Sciences, University of Rzeszow, Pigonia 1, 35-310 Rzeszow, Poland; alewinska@ur.edu.pl (A.L.); irzeszutek@ur.edu.pl (I.R.); boklejewicz@ur.edu.pl (B.O.); eszpyrka@ur.edu.pl (E.S.); 3 Center for Microelectronics and Nanotechnology, Institute of Materials Engineering, University of Rzeszow, Pigonia 1, 35-310 Rzeszow, Poland; rwojnarowska@ur.edu.pl; 4 Faculty of Chemistry, University of Warsaw, Pasteura 1, 02-093 Warsaw, Poland; a.krogul@uw.edu.pl; 5 CITAB—Centre for the Research and Technology of Agro Environment and Biological Sciences, University of Trás-os-Montes and Alto Douro, 5000-801 Vila Real, Portugal; alfredoa@utad.pt; 6 Higher Institute of Applied Biological Sciences of Tunis, University of Tunis El Manar, Tunis 1068, Tunisia

**Keywords:** pearl millet, encapsulated polyphenolic extract, breast cancer, apoptosis, senescence

## Abstract

**Simple Summary:**

In the present study, anticancer activity of pearl millet polyphenol-rich extract and encapsulated pearl millet polyphenol-rich fraction was investigated and compared using three cellular models of breast cancer in vitro. Proliferating and non-proliferating (drug-induced senescent) breast cancer cells were considered. Non-tumorigenic corresponding cells were used as control cells. Microencapsulation sensitized breast cancer cells to the treatment with pearl millet-based preparation. Cytotoxic effects were mediated by the induction of apoptotic cell death in the three breast cancer cell lines used. In one breast cancer cell line, encapsulated pearl millet extract also promoted cytotoxic autophagy. The usefulness of the microencapsulation approach was documented, especially in terms of the augmentation of anticancer effects of plant-derived compounds with limited bioavailability.

**Abstract:**

Plant-derived polyphenols are bioactive compounds with potential health-promoting properties including antioxidant, anti-inflammatory, and anticancer activity. However, their beneficial effects and biomedical applications may be limited due to their low bioavailability. In the present study, we have considered a microencapsulation-based drug delivery system to investigate the anticancer effects of polyphenol-rich (apigenin, caffeic acid, and luteolin) fractions, extracted from a cereal crop pearl millet (*Pennisetum glaucum*), using three phenotypically different cellular models of breast cancer in vitro, namely triple negative HCC1806, ER-positive HCC1428, and HER2-positive AU565 cells. Encapsulated polyphenolic extract induced apoptotic cell death in breast cancer cells with different receptor status, whereas it was ineffective against non-tumorigenic MCF10F cells. Encapsulated polyphenolic extract was also found to be cytotoxic against drug-resistant doxorubicin-induced senescent breast cancer cells that were accompanied by increased levels of apoptotic and necrotic markers, cell cycle inhibitor p21 and proinflammatory cytokine IL8. Furthermore, diverse responses to the stimulation with encapsulated polyphenolic extract in senescent breast cancer cells were observed, as in the encapsulated polyphenolic extract-treated non-proliferating AU565 cells, the autophagic pathway, here cytotoxic autophagy, was also induced, as judged by elevated levels of beclin-1 and LC3b. We show for the first time the anti-breast cancer activity of encapsulated polyphenolic extract of pearl millet and postulate that microencapsulation may be a useful approach for potentiating the anticancer effects of phytochemicals with limited bioavailability.

## 1. Introduction

Female breast cancer is reported to be the most frequently diagnosed cancer worldwide [1,2]. Despite the fact that there are numerous therapeutic options available based on breast cancer subtype, namely surgery along with conventional chemotherapy, hormone therapy, targeted therapy, and immunotherapy, breast cancer is still the second leading cause of cancer-related deaths [1,2]. Limited treatment success may rely on the complexity and heterogeneity of breast cancer, a group of diseases with a plethora of clinical features reflecting histological, biochemical, genetic, and epigenetic traits that are not fully established yet [2]. The biology of breast cancer and novel anticancer strategies (e.g., new drugs and drug delivery systems) are initially studied using cellular models of breast cancer in vitro, namely well-established breast cancer cell lines [3]. Breast cancer cell lines are considered as an infinite source of a homogeneous cell populations reflecting the main clinical types of breast cancer based on receptor status, e.g., estrogen receptor (ER)-positive, human epidermal growth factor receptor 2 (HER2)-positive, and triple negative (TNBC, ER-negative, progesterone receptor (PR)-negative, and HER2-negative) subtypes [3]. As TNBC is not sensitive to endocrine therapy or HER2-based targeted therapy and is characterized by high invasiveness and poor prognosis, the development of new TNBC treatment strategies is urgently needed [4,5].

Polyphenols are ubiquitously present in plant-based foods and beverages such as vegetables, fruits, cereals, tea, and red wine, and a polyphenol-rich diet is suggested to promote beneficial health effects, including anticancer activity [6,7]. Mechanistically, polyphenol-mediated anticancer action is based on the interference with cancer-driving signaling pathways associated with the aberrant activity of PI3K, Akt, mTOR, RAS, MAPK, NF-κB, and p53 [8]. Selected polyphenols such as resveratrol, quercetin, kaempferol, genistein, epigallocatechin-3-gallate, apigenin, fisetin, and luteolin were found to be active against TNBC, as judged by their ability to inhibit cell proliferation, stimulate apoptosis, and suppress cell migration and invasion in preclinical models of breast cancer in vitro and in vivo [9,10]. Furthermore, polyphenols are often reported as compounds that can potentiate the action of chemotherapeutic drugs and suggested to be promising candidates for combination therapy [9]. However, their poor water solubility and rapid metabolism may result in low bioavailability in an in vivo situation, which may in turn limit their therapeutic efficacy [10,11]. To overcome such obstacles, several nano-carriers have been developed, such as the application of polymeric, lipid-based, metallic, magnetic, and inorganic nanoparticles, nanogels, nanoliposomes, nanocrystals, and electrospun nano-fibers to ameliorate the solubility, stability, efficiency, and safety profiles of polyphenols in biological systems [12,13,14,15,16]. As initial results are promising in terms of improved pharmacokinetics and pharmacodynamics, more studies are needed to document the usefulness of nano-polyphenols in anticancer therapies, especially TNBC treatment.

Pearl millet (*Pennisetum glaucum* (L.) R. Br., the *Poaceae* family) is one of the most important drought-tolerant and pest-resistant C4 cereal crop cultivated for forage, grain, and stover, mainly in the tropics and sub-tropics of sub-Saharan Africa (e.g., Niger, Mali, Nigeria) and Asia (e.g., India) [17,18,19,20]. Millets serve as staple foods for millions of people in the developing countries, being a rich source of nutrients and bioactive phytochemicals such as phenolic compounds with prospective health benefits [17,18,19,20]. As millets are underutilized grasses in the developed countries, it is worthwhile analyzing the chemical composition and investigating the biological activity of millet grains in more detail.

In the present study, we have designed and tested a microencapsulation-based drug delivery system to treat breast cancer cells with different receptor status (triple negative HCC1806, ER-positive HCC1428, and HER2-positive AU565 cells) with polyphenol-rich fraction (apigenin, caffeic acid, and luteolin) derived from pearl millet grains. Encapsulated polyphenolic extract was found to be active against both proliferating and drug-induced senescent breast cancer cells and TNBC cells were the most sensitive to the treatment with encapsulated polyphenolic extract. Diverse cell death responses were revealed and discussed.

## 2. Materials and Methods

### 2.1. Preparation of Pearl Millet Extract

Pearl millet (PM) was cultivated in the region of Mateur (North Tunisia). The grain of PM was collected, air-dried in the dark, and transformed into powder using an electric blender. For the extraction of polyphenols, 20 g of PM grain powder was extracted two times (each lasting 2 h) with 400 mL of methanol–acetone–water (7:7:6, *v*/*v*/*v*) at room temperature (25 °C) with continuous stirring. After extraction, the mixture was centrifuged (4000× *g*, 20 min) (5804R, Eppendorf, Hamburg, Germany) and the supernatant was collected and concentrated using a rotary evaporator at 60 °C (Laborta 4000 Heidolph, Schwabach, Germany) [21]. PM residues were dissolved in dimethyl sulfoxide (DMSO), while the remaining residues were re-dissolved in 100% ethanol. The resulting dry residues were weighed and used for further analysis.

### 2.2. Lipid Extraction

Lipids were extracted according to the method of Nani et al. (2015) [21]. The PM powder (20 g) was blended with 160 mL of isopropanol and heated at 80 °C for 5 min. It was then cooled to room temperature and dissolved in 240 mL of hexane. The mixture was centrifuged, and then the upper phase was collected. The pellet was re-extracted with 180 mL of hexane and 40 mL of isopropanol. This extract was combined with the upper phase from the previous steps. To remove the non-lipidic fraction, the extract was partitioned into an upper hexane phase by adding aqueous sodium sulfate 6.5% (0.5/1, *v*/*v*). The resulting upper phase, the lipid extract, was transferred into a new tube. The extract was stored at −20 °C.

### 2.3. HPLC Analysis

The profile and content of polyphenols in PM samples were analyzed using HPLC-diode array detector (DAD) system, as described comprehensively elsewhere [22]. Briefly, 500 µL of the extract was used and 0.1% of trifluoroacetic acid (TFA) and acetonitrile with 0.1% TFA were considered as solvents. The injection volume was 10 µL and a C18 column (250 × 46 mm, 5 mm, ACE HPLC Columns, Advanced Chromatography Technologies Ltd., Abeerden, Scotland, UK) was applied. Chromatograms were recorded at 320 nm (cinnamic acids) and 370 nm (flavonoids). Polyphenol identification was based on peak retention time, UV spectra, and UV max absorbance bands, in comparison with literature and commercial external standards, and were injected simultaneously with plant extracts (Extrasynthese, Genay, France). The relative factor response and internal standard (naringin) method was used to calculate the content of individual polyphenols. The results are presented as µg per g dry weight (DW).

### 2.4. Preparation of Microcapsules

To prepare the microcapsules, tryptone soya agar (TSA) with lecithin and TWEEN^®^80 (T3813, Merck KGaA, Darmstadt, Germany), glycerol (G2025, Merck KGaA, Darmstadt, Germany), and food-grade cold-pressed linseed oil (ZT Kruszwica S.A., Kruszwica, Poland) were used. TSA was mixed with linseed oil in a ratio of 2:5, and the mixture was then heated at 40 °C for 40 min to dissolve TSA. A total of 60 µg/mL polyphenol extract (dissolved in DMSO or EtOH) and 0.5% glycerol solution were then added. Optionally, 10 µM fluorescein was also added. The mixture was stirred at 40 °C for 1 h. Afterward, the mixture was subjected to ultrasonication for a total time of 1 h (5 min ultrasonication and 5 min non-ultrasonication intervals). The following parameters were considered: frequency: 45 kHz, ultrasonic power: 150 W, and temperature: 25 °C. The resulting mixture was then purged with nitrogen for 20 min to stabilize the obtained microcapsules.

### 2.5. Dynamic Light Scattering (DLS)

The size distribution of microcapsules was analyzed using dynamic light scattering (DLS) instrument Zetasizer Nano-ZS (Malvern Instruments, Malvern, UK). The sample (1 mL) was placed in a polystyrene cuvette (10 × 10 × 45 mm, cell type DTS0012) and data were collected at a 173° backscatter angle. DLS measurements were conducted using polarized laser light with a wavelength of 632.8 nm, at 25 °C, pH = 7.0, for concentration range of samples from 0.62 to 2.50%. The results are calculated as an average of three measurements consisting of 11 runs for a period of 10 s.

### 2.6. Raman Spectroscopy

The Raman spectra were recorded using an inVia Micro Raman Renishaw spectrometer combined with a Leica DM 2500M microscope (Renishaw, Gloucestershire, UK). The spectrometer is equipped with a 785 nm laser as an excitation source. The measurements were taken with an exposure time of 10 s with five times scan accumulation and for the laser output power of 10 mW. The data were collected in the spectral range of 200–4000 cm^−1^. The measurements were carried out with a 100× lens magnification. Baseline correction and normalization were performed during data processing.

### 2.7. Analysis of Total Polyphenol Content Using Folin–Ciocalteu Assay

Microcapsules with and without polyphenolic extracts (60 µL) were mixed with freshly prepared 300 µL of Folin–Ciocalteu reagent diluted in H_2_O (1:10) and 240 µL of 7.5% Na_2_CO_3_. The mixture was incubated at 45 °C for 15 min in the dark. The samples were then centrifuged and the absorbance of the supernatants was read at 765 nm using UV–Vis Spectrophotometer (Infinite M200 Pro, Tecan, Männedorf, Switzerland) against a blank (deionized water). Total polyphenol content was calculated on the basis of a calibration curve obtained for a gallic acid solution. Results are expressed in mg of gallic acid equivalents, GAE per g dry extract.

### 2.8. Protection against Fluorescein Bleaching Test

The scavenging activity of polyphenols present in encapsulated polyphenolic extract was evaluated as the ability to protect against oxidant-mediated fluorescein bleaching. Three types of microcapsules containing fluorescein were considered, namely empty microcapsules (MC*), microcapsules with polyphenolic extract dissolved in DMSO (PDMC*), and microcapsules with polyphenolic extract dissolved in ethanol (PEMC*). Briefly, 1386 µL of 2.5% microcapsules (in PBS) was added to 14 µL of hydrogen peroxide (H_2_O_2_, final concentration of 100 µM) and incubated for 10 min in the dark. The fluorescence in fluorescence relative units (RFU) was measured at λ_ex_ = 450 nm and λ_em_ = 514 nm using a fluorescence microplate reader (Infinite M200 Pro, Tecan, Männedorf, Switzerland). The results were normalized to non-treated control.

### 2.9. Cell Lines and Culture Conditions

The following breast cancer cell lines, namely triple negative breast cancer (TNBC) HCC1806 (CRL-2335^™^), ER-positive HCC1428 (CRL-2327^™^), and HER2-positive AU565 (CRL-2351^™^) (ATCC, Manassas, VA, USA), were grown in RPMI-1640 medium (Corning, Tewksbury, MA, USA) supplemented with 10% FBS and antibiotics (100 U/mL penicillin, 0.1 mg/mL streptomycin, and 0.25 g/mL amphotericin). MCF10F non-tumorigenic epithelial cells from mammary gland (CRL-10318^™^, ATCC, Manassas, VA, USA) were cultivated in DMEM/F12 medium (Merck KGaA, Darmstadt, Germany) containing 5% horse serum, 10 ng/mL epidermal growth factor EGF, 10 µg/mL insulin, 0.5 µg/mL hydrocortisone, 100 ng/mL cholera toxin, and mix of antibiotics. Cells were cultured at 37 °C in a controlled humidified atmosphere containing 5% CO_2_ and passaged with trypsin solution (0.05% trypsin for MCF10F cells and 0.25% trypsin for breast cancer cells, respectively, Corning, Tewksbury, MA, USA).

### 2.10. MTT Assay

The effects of free pearl millet extracts in DMSO (PD), ethanol (PE), and lipids (PL), and encapsulated PD (PDMC) and PE (PEMC) on the metabolic activity of normal and breast cancer cells were investigated using the MTT test [23]. Briefly, upon 24 h culture (seeded density of 10,000 cells per well of a 96-well plate), cells were treated with 10, 20, 40, and 60 µg/mL PD, PE, and PL as well as 0.62%, 1.25%, and 2.5% empty microcapsules (MC), PDMC, and PEMC for 24 h. The effects of solvents (DMSO, ethanol) were also considered. Untreated control conditions served as the baseline, with a normalization of 100%.

### 2.11. Encapsulated Polyphenolic Extract-Mediated Apoptotic Cell Death

Microcapsule-induced apoptosis was studied in both proliferating (normal and cancer) and non-proliferating (drug-induced senescent) cancer cells. To stimulate therapy-induced senescence (TIS), breast cancer cells were treated with 100 nM doxorubicin (Merck KGaA, Darmstadt, Germany) for 24 h and cultured for up to 7 days after drug removal. A portion of fresh medium was added every 2 days to avoid nutrient limitation. The activation of the drug-induced senescence program was confirmed using senescence-associated β-galactosidase activity test (CellEvent™ Senescence Green Detection Kit, Thermo Fisher Scientific, Waltham, MA, USA) and imaging flow cytometry (Amnis^®^ FlowSight^®^ imaging flow cytometer, Luminex Corporation, Austin, TX, USA) according to the manufacturer’s instructions. Proliferating and non-proliferating cells were then treated with the microcapsules (0.62% and/or 2.5%) for 24 h. Several markers of apoptotic cell death were considered, namely phosphatidylserine externalization and the levels of caspase 9 (an initiator caspase and a marker of mitochondrial pathway of apoptosis) and caspase 3 (a main executioner caspase in both intrinsic and extrinsic apoptotic pathways). Phosphatidylserine externalization was analyzed using the Muse^®^ Cell Analyzer and the Muse^®^ Annexin V and Dead Cell Assay Kit (Luminex Corporation, Austin, TX, USA), as previously described [24]. Briefly, cells were subjected to dual staining of Annexin V staining (apoptosis evaluation) and 7-AAD staining (necrosis evaluation) and four sub-populations were analyzed: live cells (Annexin V-negative, 7-AAD-negative), early apoptotic cells (Annexin V-positive, 7-AAD-negative), late apoptotic cells (Annexin V-positive, 7-AAD-positive), and necrotic cells (Annexin V-negative, 7-AAD-positive). Representative dot-plots (%) with corresponding quantitative analysis are presented. Furthermore, the levels of caspase 9 and caspase 3 were assayed in fixed cells using dedicated antibodies and imaging cytometry. Briefly, primary antibodies anti-caspase-3 (1:140, PA5-77887) and anti-caspase-9 (1:100, PA5-17913), and secondary antibodies conjugated with fluorochromes (anti-rabbit secondary antibody conjugated with Texas Red (1:1000, T2767), and anti-rabbit secondary antibody conjugated with FITC (1:1000, T2765) (Thermo Fisher Scientific, Waltham, MA, USA)) were used and fluorescence signals were analyzed using a confocal imaging system IN Cell Analyzer 6500 HS and IN Carta software 1.14 (Cytiva, Marlborough, MA, USA). The levels of caspase 9 and caspase 3 are presented in relative fluorescence units (RFU).

### 2.12. The Analysis of Microcapsule Uptake and Encapsulated Polyphenolic Extract-Mediated Antiproliferative, Immunomodulatory, and Autophagy-Inducing Effects in Senescent Breast Cancer Cells

Doxorubicin-induced senescent breast cancer cells were treated with 0.62% encapsulated polyphenolic extracts (without fluorescein) for 24 h, fixed, and the levels of cell cycle inhibitor p21, pro-inflammatory cytokine IL8, and autophagic markers beclin-1 and LC3b were then evaluated using dedicated antibodies, namely primary antibodies anti-p21 (1:800, MA5-14949), anti-IL8 (1:500, ab154390), anti-BECN1 (1:100, TA502527), and anti-LC3b (1:500, PA5-32254), and secondary antibodies conjugated with fluorochromes (anti-rabbit secondary antibody conjugated with Texas Red (1:1000, T2767), anti-rabbit secondary antibody conjugated with FITC (1:1000, T2765), and anti-mouse secondary antibody conjugated with cyanine 5 (1:1000, A10524) (Thermo Fisher Scientific, Waltham, MA, USA, Abcam, Cambridge, UK)). Furthermore, using encapsulated polyphenolic extract containing fluorescein, microcapsule uptake was also tracked as increased intracellular fluorescence signals. Fluorescence signals were analyzed using a confocal imaging system IN Cell Analyzer 6500 HS and IN Carta software 1.14 (Cytiva, Marlborough, MA, USA). The uptake of microcapsules and the levels of p21, IL8, beclin-1, and LC3b are presented in relative fluorescence units (RFU).

### 2.13. Statistical Analysis

The results are presented as mean ± standard deviation (SD) of three replicates. For imaging cytometry-based data, box and whisker plots with median, lowest, and highest values were also considered. Microcapsule-treated and non-treated cells were compared using one-way analysis of variance (ANOVA and Dunnett’s multiple comparison test), while PDMC and PEMC treatment was compared to MC treatment (ANOVA and Tukey’s a posteriori test). For analyzing the data on the encapsulated polyphenolic extract-mediated protection against oxidant-induced fluorescein bleaching, Student’s *t*-test was also used. *p*-values of less than 0.05 were considered as statistically significant. Statistical analysis was conducted using the GraphPad Prism 8 software.

## 3. Results and Discussion

### 3.1. Polyphenol Content and Initial Analysis of Anti-Breast Cancer Effects of Pearl Millet Grain Extracts

The results of polyphenol content of pearl millet grain ethanolic extracts by HPLC-DAD are presented in Figure S1 and six phenolics grouped into three major classes were identified, namely hydroxybenzoic acids (vanillic acid and syringic acid), hydroxycinnamic acids (coumaric acid, caffeic acid and its derivatives), and flavones (apigenin and luteolin). The most abundant individual phenolics were apigenin (24.551 µg per g DW), caffeic acid (24.023 µg per g DW) and luteolin (15.341 µg per g DW). The total phenolics were 46.73 µg per g dry weight of soluble phenolics and 31.02 µg per g of insoluble phenolics. Our data are in agreement with previously published data on the major polyphenols that can be found in pearl millet grains [17]. For example, the most abundant polyphenol-based phytochemicals detected in pearl millet were hydroxybenzoic acids and their derivatives (*p*-hydroxybenzoic, protocatechuic, and vanillic acids), hydroxycinnamic acids and their derivatives (caffeic, *p*-coumaric, cinnamic, sinapic, and trans-ferulic acids), and flavonoids (apigenin and myricetin) [17]. Polyphenols found in millets are suggested to exert several health promoting effects such as antioxidant, antihypertensive, hypocholesterolemic, hypoglycemic, immunomodulatory, antibacterial, antimicrobial, and anticancer effects [17,18,20]. Nonetheless, the anticancer potential of pearl millet-based preparations has never been assessed.

**Figure 1:**
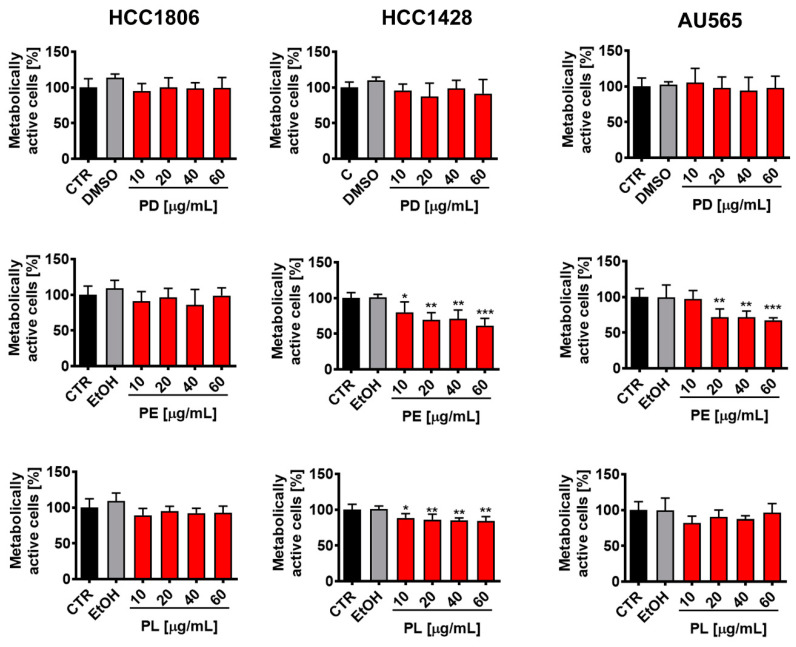
The effects of polyphenolic fractions dissolved in DMSO (PD, (**top**)), ethanol (PE, (**middle**)), and lipids (PL, (**bottom**)) extracted from pearl millet grains on metabolic activity of three breast cancer cell lines, namely triple negative HCC1806, ER-positive HCC1428, and HER2-positive AU565 cells. Cells were treated with pearl millet extracts at the concentrations of 10, 20, 40, and 60 μg/mL for 24 h. Metabolic activity was assayed using MTT test. Untreated control (CTR) is considered as 100%. The effects of solvents (DMSO, EtOH) were also tested. Bars indicate SD, *n* = 6, *** *p* < 0.001, ** *p* < 0.01, * *p* < 0.05 compared to CTR (ANOVA and Dunnett’s a posteriori test).

Polyphenol content in fractions dissolved in DMSO (PD), ethanol (PE), and lipid extracts (PL) was then tested against three breast cancer cell lines with different mutation and receptor status (triple negative HCC1806, ER-positive HCC1428, and HER2-positive AU565) [3] using MTT assay (Figure 1). A range of extract concentrations from 10 to 60 µg/mL and 24 h exposure time were considered. At lower concentrations, extracts were not effective and at higher concentrations, we have also observed the effects of solvents used. Thus, this range of concentrations was selected for the analysis. We have also considered the low bioavailability of phytochemicals in biological systems according to previously published data and decided not to use high concentrations of extracts that could not be achieved in the in vivo situation (higher than 60 µg/mL). PD did not affect the metabolic activity of three breast cancer cell lines, and PL-mediated effects were slight and limited to HCC1428 cells, whereas the effects of PE were the most pronounced (Figure 1). PE promoted a statistically significant decrease in metabolic activity of HCC1428 and AU565 cells compared to untreated corresponding controls (CTR) (Figure 1). The effects of solvents used (DMSO, EtOH) were also ruled out (Figure 1). Triple negative HCC1806 cells were insensitive to extract treatments (PD, PE, and PL) (Figure 1). As 60 µg/mL PE was the most effective in terms of inhibiting the metabolic activity of HCC1428 and AU565 cells (a decrease in metabolic activity to about 60% of control levels) (Figure 1), this concentration was selected for the preparation of encapsulated polyphenolic extracts. Data on anticancer effects of millets are scarce and based mainly on the use of one cancer cell line [25,26]. For example, anticancer activity of elephant grass (*Pennisetum purpureum* Schumach.) methanolic extract (whole plant) was analyzed using leukemia CCRF-CEM cells and resazurin assay-based changes in metabolic activity [25]. After 72 h treatment with elephant grass extract, the IC_50_ value was established to be 69.01 ± 7.99 µg/mL [25]. The authors did not focus on elephant grass-associated mode of cell death and related mechanisms in leukemic cells [25]. Foxtail millet bran-based preparation in a form of bound polyphenols of the inner shell (BPIS, *Setaria italica*) was found to induce apoptotic cell death in colorectal cancer HCT-116 cells [26]. However, the anticancer (pro-apoptotic) effects of BPIS were revealed at high concentration of 600 µg/mL [26].

### 3.2. Physico-Chemical Characterization of Microcapsules

**Figure 2:**
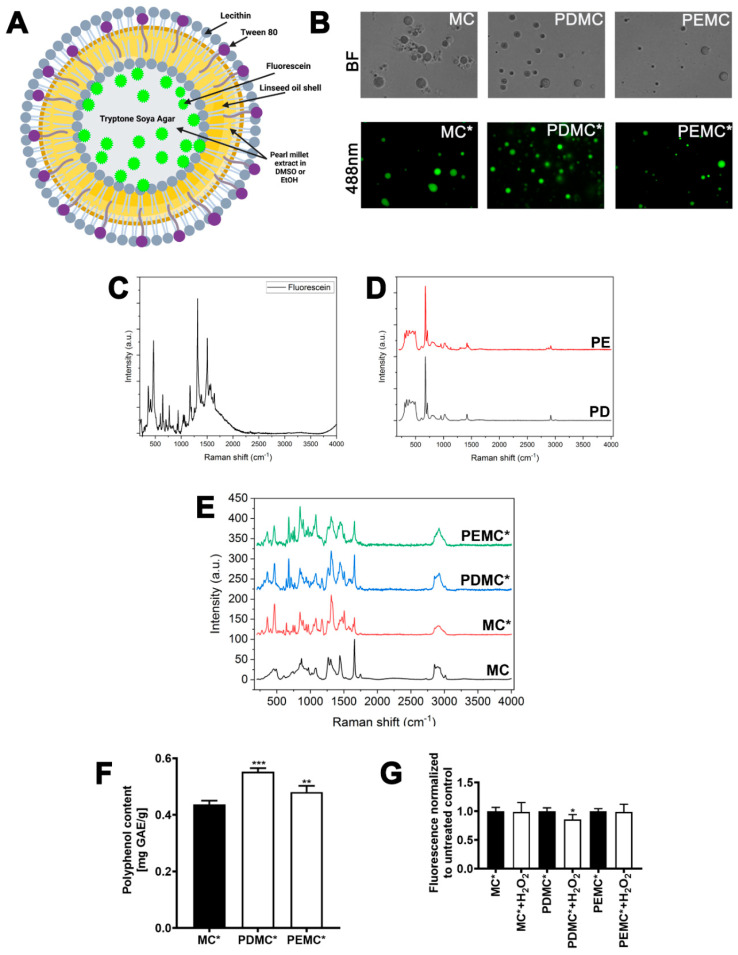
Physico-chemical characterization of empty microcapsules (MC), microcapsules containing polyphenols extracted in DMSO (PDMC), and microcapsules containing polyphenols extracted in ethanol (PEMC). For selected experiments, microcapsules containing fluorescein were also used and are denoted with an asterisk (*). (**A**) A schematic graph showing the main components of prepared microcapsules. (**B**) Representative microphotographs showing the morphology of microcapsules not containing fluorescein (MC, PDMC, and PEMC, upper panel, bright field, BF) and microcapsules containing fluorescein (MC*, PDMC*, and PEMC*, lower panel, FITC fluorescence filter, 488 nm), objective 40×. (**C**) Raman spectra of fluorescein. (**D**) Raman spectra of polyphenols in PD and PE extracts. (**E**) Raman spectra of PEMC*, PDMC*, MC*, and MC. (**F**) The total polyphenol content in MC*, PDMC*, and PEMC*. The total polyphenol content was analyzed using Folin–Ciocalteu assay. The total polyphenol content is expressed as mg GAE per g. Bars indicate SD, *n* = 6, *** *p* < 0.001, ** *p* < 0.01 compared to empty microcapsules containing fluorescein (MC*) (ANOVA and Dunnett’s a posteriori test). (**G**) Protection of hydrogen peroxide-mediated fluorescein bleaching by encapsulated polyphenolic extracts PDMC* and PEMC* containing fluorescein. Empty microcapsules containing fluorescein (MC*) served as a control. Microcapsules containing fluorescein were treated with hydrogen peroxide and fluorescein-based fluorescence was then measured. Fluorescence in relative fluorescent units was normalized to corresponding untreated control. Bars indicate SD, *n* = 6, * *p* < 0.05 compared to corresponding untreated control (Student’s *t*-test).

The polyphenolic extracts dissolved either in DMSO (PD) or EtOH (PE) were immobilized in the microcapsules to obtain PD-based and PE-based microcapsules, PDMC and PEMC, respectively (Figure 2A). The control empty microcapsules (MC) were also considered. The microcapsules were prepared using linseed oil, lecithin, Tween 80, tryptone soya agar (TSA), and glycerol (Figure 2A). To track the cellular uptake of microcapsules, the microcapsules containing fluorescein were also prepared (MC*, PDMC*, and PEMC*) (Figure 2A,B). DLS-based analysis revealed that microcapsule preparations (0.62, 1.25, and 2.5%) were polydisperse, as microcapsule size, expressed as a hydrodynamic diameter, ranged from 25 to 250 nm (Figure S2).

Raman spectroscopy was then used to characterize pearl millet extracts and corresponding microcapsules. The Raman spectra of fluorescein, polyphenolic fractions dissolved in DMSO (PD) and EtOH (PE), encapsulated polyphenolic extracts containing fluorescein (PDMC* and PEMC*), and control empty microcapsules with (MC*) and without (MC) fluorescein are presented in Figure 2C, D and E, respectively. A number of lines attributed to the vibrations of the structures and chemical bonds of individual molecules (marker bands) were detected. Fluorescein-based marker lines are spectral bands at 467 cm^−1^, 643 cm^−1^, and 1317 cm^−1^ (Figure 2C). For polyphenols, marker lines are spectral bands at 674 cm^−1^ and 710 cm^−1^, attributed to stretch ring vibrations (Figure 2D) [27,28]. Based on the spectrum for the MC sample, spectral bands characteristic of prepared microcapsules were established: 453 cm^−1^, 493 cm^−1^, 604 cm^−1^, 742 cm^−1^, 844 cm^−1^, 867 cm^−1^, 891 cm^−1^, 972 cm^−1^, 1083 cm^−1^, 1265 cm^−1^, 1305 cm^−1^, 1444 cm^−1^, 1659 cm^−1^, 1747 cm^−1^, 2853 cm^−1^, 2890 cm^−1^, 2937 cm^−1^, and 3015 cm^−1^ (Figure 2E). These signals are mainly assignable to the CCC, CC, CCH, OCO, COH, CCO, CO, CH, CH_2_, and CH_3_ vibrations of TSA [29] and to the CH, CH_2_, C=C–H, C=C, and C=O vibrations of linseed oil [30]. In the sample MC* spectra, the bands derived from the structure of the microcapsules are observed as well as the marker lines attributed to fluorescein vibrations (Figure 2E). In addition to the mentioned marker lines (467 cm^−1^, 643 cm^−1^, and 1317 cm^−1^), several weaker fluorescein bands were also noticed: 359 cm^−1^, 406 cm^−1^, 768 cm^−1^, 938 cm^−1^, 1171 cm^−1^, and 1506 cm^−1^ (Figure 2E). Slight shifts of some strands (359 cm^−1^, 938 cm^−1^, and 1312 cm^−1^ with 7 cm^−1^, 3 cm^−1^, and 5 cm^−1^ shifts, respectively) were also observed, which may indicate the interactions between fluorescein and microcapsules (Figure 2E). The analysis of Raman spectra of samples PEMC* and PDMC* revealed the bands attributed to vibrations of microcapsule shell components as well as fluorescein and polyphenol molecules (Figure 2E). The bands at 358 cm^−1^, 410 cm^−1^, 464 cm^−1^, 643 cm^−1^, 765 cm^−1^, 938 cm^−1^, 1171 cm^−1^, 1311 cm^−1^, and 1506 cm^−1^ were assigned to fluorescein (Figure 2E). The bands at 307 cm^−1^, 677 cm^−1^, 710 cm^−1^, 1417 cm^−1^, and 2922 cm^−1^ are related to polyphenols (Figure 2E) [27,28]. The slight shifts of the bands corresponding to fluorescein and polyphenols were also observed for PEMC* and PDMC*, caused by a change in the local environment of the vibrating bonds (Figure 2E). Raman vibrational frequencies of the studied samples are also listed in Table S1.

The total phenolic content in encapsulated polyphenolic extracts was also analyzed using the Folin–Ciocalteu method (Figure 2F). As expected, the encapsulated polyphenolic extracts PDMC* and PEMC* were characterized by higher levels of total phenolics (mg GAE/g) compared to empty microcapsules (MC*) (Figure 2F). Furthermore, PDMC* were more polyphenol-rich than PEMC* (Figure 2F).

The scavenging activity of encapsulated polyphenolic extracts was then tested using microcapsules containing fluorescein and hydrogen peroxide-mediated fluorescein bleaching test using cell-free system in vitro (Figure 2G). The addition of an oxidant, here hydrogen peroxide, did not affect the fluorescein-based fluorescence in the case of PEMC*, whereas a slight but statistically significant decrease in fluorescein-based fluorescence was observed when hydrogen peroxide was added to PDMC* (Figure 2G). This may suggest that the antioxidant activity of polyphenols dissolved in DMSO is slightly limited compared to the antioxidant activity of polyphenols dissolved in EtOH (Figure 2G). Furthermore, empty microcapsules containing fluorescein MC* also protected against hydrogen peroxide-mediated fluorescein bleaching (Figure 2G). One can conclude that polyphenols present in the shell of MC* (Figure 2F) may account for observed antioxidant activity of MC* (Figure 2G). We also suggest that apigenin and luteolin, two of the most abundant flavonoids in polyphenol-rich extracts of pearl millet (Figure S1), might be responsible for extract-associated antioxidant activity (Figure 2G). This result is in agreement with our previous report on apigenin- and luteolin-mediated protection against hypochlorite-induced fluorescein bleaching [31]. Polyphenol-mediated antioxidant activity of different millet grains is widely documented using a number of cell-free test systems in vitro, such as DPPH reduction capacity and ferric reducing antioxidant potential tests [17,18]. One can conclude that polyphenol-rich extracts of millet grains have free radical scavenging activity, reducing power activity, ferrous chelating properties, and inhibitory activity against the production of reactive oxygen species (ROS) in vitro [17].

### 3.3. The Anticancer Activity of Encapsulated Polyphenolic Extracts against Breast Cancer Cells

**Figure 3:**
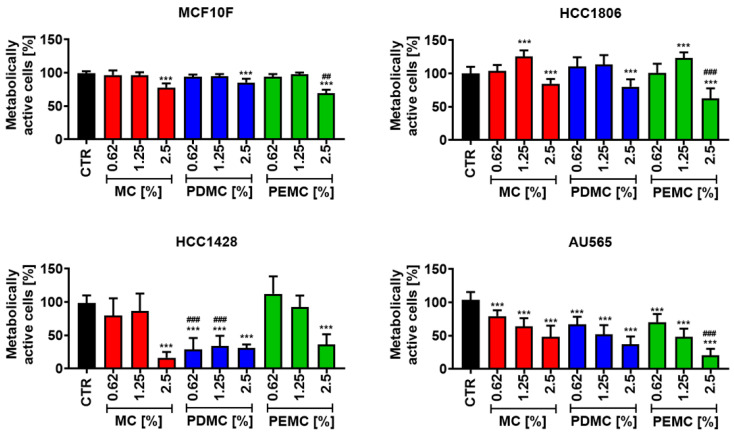
The effects of encapsulated polyphenolic extracts (PDMC and PEMC) on metabolic activity of three breast cancer cell lines, namely HCC1806, HCC1428, and AU565, and corresponding non-tumorigenic MCF10F cells. Metabolic activity was analyzed using MTT test. Cells were treated with pearl millet-encapsulated polyphenolic extracts at the concentrations of 0.62, 1.25, and 2.5% for 24 h. The effects of microcapsule-based drug delivery system (empty MC) were also tested. The metabolic activity of untreated control (CTR) is considered as 100%. Bars indicate SD, *n* = 6, *** *p* < 0.001 compared to CTR (ANOVA and Dunnett’s a posteriori test), ^###^ *p* < 0.001, ^##^ *p* < 0.01 compared to MC treatment (ANOVA and Tukey’s a posteriori test). CTR, control conditions; MC, treatment with empty microcapsules; PDMC, treatment with encapsulated pearl millet polyphenolic extract dissolved in DMSO; PEMC, treatment with encapsulated pearl millet polyphenolic extract dissolved in EtOH.

The activity of immobilized polyphenolic extracts PDMC and PEMC (60 µg/mL) was then evaluated against three breast cancer cell lines at three concentrations of microcapsules, namely 0.62, 1.25, and 2.5% (Figure 3). We have considered 24 h treatment, as microcapsules might not be stable when incubated for prolonged time in the cell culture medium. For microencapsulation, 60 µg/mL extracts were used due to observed effects against breast cancer cells. We have initially tested different concentrations of microcapsules (lower than 0.62%—have no effects, higher than 2.5%—have more side effects in terms of empty microcapsules). Thus, we have used 0.62, 1.25, and 2.5% microcapsules in the present study. The initial screening using MTT assay indicated that the microencapsulation approach potentiated the anticancer effects of pearl millet polyphenolic extracts (Figure 3) compared to free extracts (Figure 1). Free PD was not effective against three breast cancer cell lines (Figure 1), while its encapsulation resulted in statistically significant decrease in metabolic activity of all breast cancer cell lines and HCC1428 cells were the most sensitive to PDMC treatment (Figure 3). Furthermore, HCC1806 cells were sensitized to PE treatment in the form of PEMC (Figure 3). PDMC and PEMC treatment was also not effective against normal non-tumorigenic epithelial MCF10F cells (Figure 3). The minor inhibitory effects of PDMC and PEMC were only observed when microcapsules were used at the highest concentration of 2.5% (Figure 3). One can conclude that the action of PDMC and PEMC is selective to breast cancer cells. However, the microcapsule-based drug delivery system also affected the metabolic activity of treated cells (Figure 3). Cancer cells were more sensitive to MC treatment than normal corresponding cells, but these inhibitory effects, except for MC-treated AU565 cells, were limited to the highest concentration of 2.5% (Figure 3). Although this result can be considered as a side effect, the action of MC is not surprising when taking into account the total polyphenol content of the MC shell (Figure 2F).

**Figure 4:**
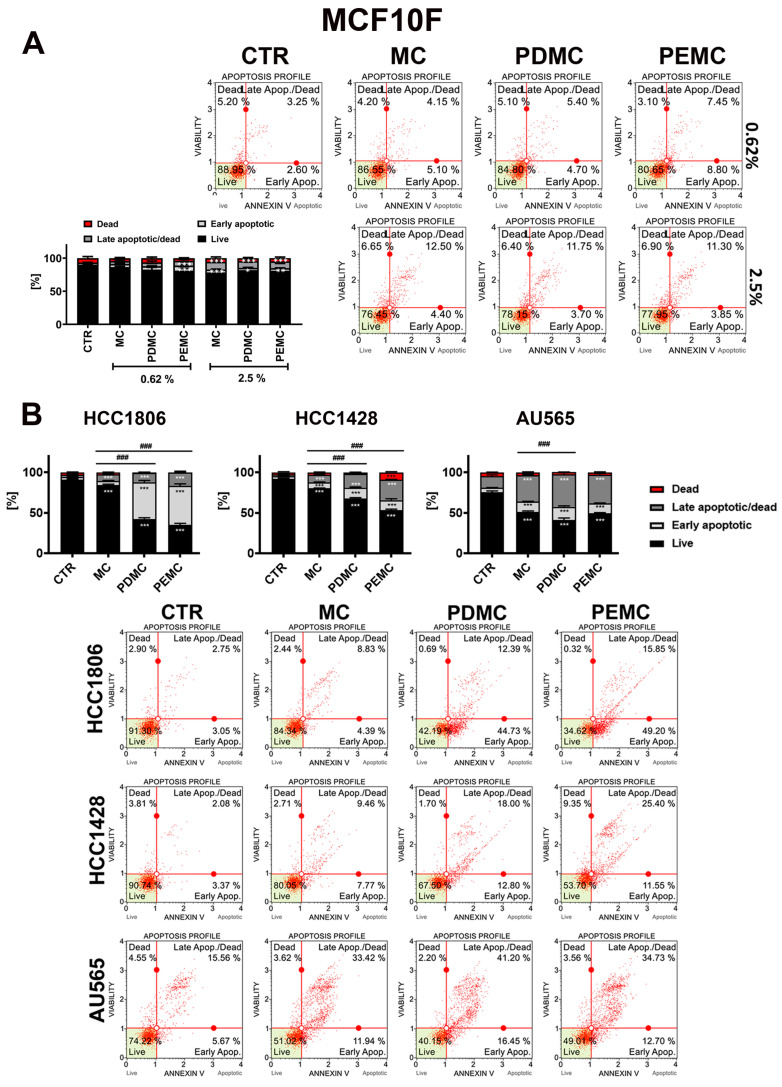
PDMC- and PEMC-mediated apoptotic cell death in three breast cancer cell lines with different receptor status, namely HCC1806, HCC1428, and AU565 (**B**) in comparison to PDMC- and PEMC-associated effects in non-tumorigenic MCF10F cells (**A**). Cells were treated with encapsulated pearl millet polyphenolic extracts at the concentrations of 0.62 and 2.5% (normal cells, (**A**)) and 2.5% (cancer cells, (**B**)) for 24 h. Apoptosis was assessed as phosphatidylserine externalization using Annexin V staining and flow cytometry. Bars indicate SD, *n* = 3, *** *p* < 0.001, ** *p* < 0.01, * *p* < 0.05 compared to CTR (ANOVA and Dunnett’s a posteriori test), ^###^ *p* < 0.001 compared to MC treatment (ANOVA and Tukey’s a posteriori test). Representative dot-plots are also shown. CTR, control conditions; MC, treatment with empty microcapsules; PDMC, treatment with encapsulated pearl millet polyphenolic extract dissolved in DMSO; PEMC, treatment with encapsulated pearl millet polyphenolic extract dissolved in EtOH.

As the MTT test is considered as an initial screening test to assess the effects of tested compounds using cell culture model in vitro without providing the information on the nature of changes in metabolic activity (e.g., the discrimination between the cytotoxic versus cytostatic effects), the ability of encapsulated pearl millet polyphenolic extracts to induce apoptotic cell death in breast cancer cells was then studied (Figure 4). Breast cancer cells were treated with 2.5% microcapsules for 24 h and phosphatidylserine externalization, a marker of apoptosis, was then assayed. Surprisingly, the least effective HCC1806 cells, according to the MTT test, were found to be the most prone to PDMC- and PEMC-induced apoptosis among breast cancer cells analyzed (Figure 4B). Except for MC-treated AU565 cells, the effects of empty microcapsules (MC) were limited. Perhaps, for HCC1806 and HCC1428 cells, a microcapsule-based drug delivery system is a good approach to enhance the anticancer effects of plant-derived polyphenols, here pearl millet polyphenolic extracts (Figure 4B). In the case of AU565 cells, the effects of PDMC and PEMC were masked due to the pro-apoptotic activity of empty microcapsules (Figure 4B). However, our developed microcapsule-based drug delivery system (MC treatment) was found to be ineffective against non-tumorigenic MCF10F cells (Figure 4A). Perhaps this may suggest that microencapsulation would not affect the viability of normal cells. Furthermore, the encapsulated polyphenolic extracts, both PDMC and PEMC, did not stimulate massive apoptosis in MCF10F cells compared to breast cancer cells (Figure 4). A slight decrease in metabolic activity was only observed in the case of the treatment with 0.62% PEMC and 2.5% PDMC and PEMC, but at the concentration of 2.5%, empty microcapsules also promoted a slight decrease in metabolic activity of normal MCF10F cells (Figure 3). This result confirmed that the action of PDMC and PEMC is selective against breast cancer cells (Figure 4). As pearl millet extracts were found to be rich in such flavonoids as apigenin and luteolin (Figure S1), one can speculate that these polyphenols may be responsible for observed anti-breast cancer effects, especially against TNBC (Figure 4). Indeed, apigenin and luteolin were reported to be active against TNBC by promoting cell cycle arrest and apoptosis and limiting cell invasion and migration that was achieved by the interference with cell survival signaling pathways [9,32]. Nanoencapsulation of apigenin has been already considered to improve its therapeutic potential [33,34]. For example, nanoencapsulation of apigenin with whey protein isolate (WPI) enhanced cellular uptake, potentiated anticancer (pro-apoptotic) activity against colorectal HCT-116 and HT-29 cancer cells in vitro, and improved in vivo bioavailability in blood and colon mucosa of tested mice compared to free apigenin treatment [33]. The usefulness of different nano-based drug delivery systems such as nanoparticles, nanocrystals, polymeric micelles, phytosomes, and liposomes was also documented for potentiating anti-breast cancer activity of apigenin [34].

### 3.4. The Anticancer Activity of Encapsulated Polyphenolic Extracts against Drug-Induced Senescent Breast Cancer Cells

**Figure 5:**
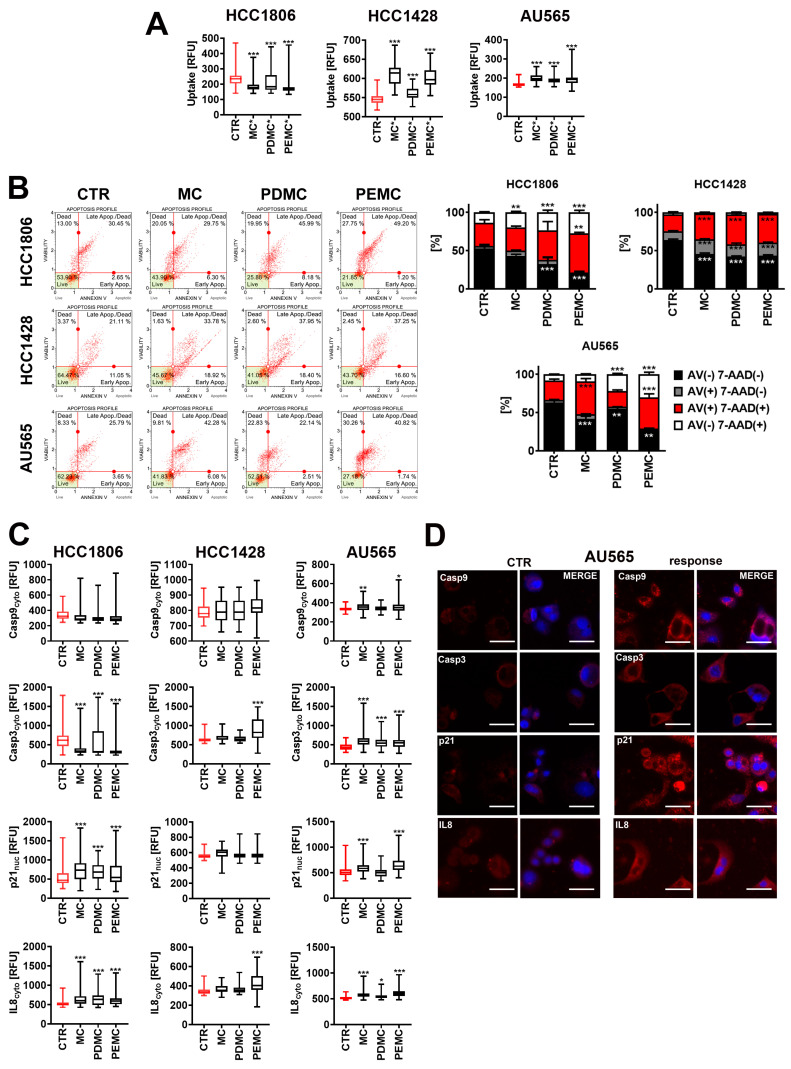
The uptake of microcapsules (**A**) and encapsulated polyphenolic extract-mediated anticancer effects in doxorubicin-induced senescent breast cancer cells HCC1806, HCC1428, and AU565 (**B**,**C**). Senescent breast cancer cells were treated with 0.62% microcapsules for 24 h. (**A**) The uptake of microcapsules was studied using fluorescein-containing microcapsules (MC*, PDMC*, and PEMC*) and imaging cytometry. The results are presented as relative fluorescence units (RFU). Box and whisker plots are shown, *n* = 6, *** *p* < 0.001 compared to CTR (ANOVA and Dunnett’s a posteriori test). (**B**) The analysis of phosphatidylserine externalization, a marker of apoptosis using Annexin V staining and flow cytometry. Bars indicate SD, *n* = 3, *** *p* < 0.001, ** *p* < 0.01 compared to CTR (ANOVA and Dunnett’s a posteriori test). Representative dot-plots are also presented. (**C**,**D**) The analysis of the levels of cytoplasmic caspase 9 (Casp9) and caspase 3 (Casp3) (apoptotic markers), nuclear p21 (cell cycle inhibitor), and cytoplasmic pro-inflammatory cytokine IL8 using dedicated antibodies and imaging cytometry. (**C**) Data are presented as relative fluorescence units (RFU). Box and whisker plots are shown, *n* = 6, *** *p* < 0.001, ** *p* < 0.01, * *p* < 0.05 compared to CTR (ANOVA and Dunnett’s a posteriori test). (**D**) Representative microphotographs of non-treated (CTR) and treated AU565 cells (response) are also shown. Casp9, Casp3, p21, and IL8 immunosignals are presented in red (immunostaining with dedicated primary antibodies and fluorochrome conjugated secondary antibodies). Nuclei are presented in blue (Hoechst 33342 staining). Objective 20×. CTR, control conditions; MC, treatment with empty microcapsules; PDMC, treatment with encapsulated pearl millet polyphenolic extract dissolved in DMSO; PEMC, treatment with encapsulated pearl millet polyphenolic extract dissolved in EtOH.

It is widely accepted that chemotherapy-induced senescent (non-proliferating) cancer cells are non-sensitive to cell death and characterized by drug resistance by limited response to apoptotic stimuli and/or upregulation of pro-survival pathways [35,36]. Thus, we decided then to test our microcapsule-based drug delivery system in senescent breast cancer cells as well. To do so, breast cancer cells were treated with a chemotherapeutic agent, doxorubicin (100 nM, 24 h stimulation), and left in the culture without the presence of the drug for additional 7 days to activate the chemotherapy-induced senescence program. Drug-induced senescence was confirmed using senescence-associated beta-galactosidase assay (Figure S3). Doxorubicin-induced senescent breast cancer cells were then incubated with encapsulated pearl millet polyphenolic extracts PDMC and PEMC for 24 h. We have considered the microcapsule concentration of 0.62%, as this concentration had no effect on metabolic activity of proliferating HCC1806 cells (Figure 3). The microcapsule concentration of 2.5% was not selected, as this concentration promoted a massive apoptotic cell death in all breast cancer cell lines (Figure 4B). First, fluorescein-containing microcapsules were used to track the uptake of microcapsules (Figure 5A). Except for senescent HCC1806 cells, an increase in fluorescein-based fluorescence was observed in all breast cancer cell lines treated with the microcapsules (Figure 5A). However, the uptake data were very variable in the case of treated HCC1806 cells (Figure 5A). Despite these discrepancies, the treatment with encapsulated polyphenolic extract in senescent breast cancer cells resulted in apoptosis induction, as judged by Annexin V staining (Figure 5B). HCC1806 senescent cells were the most sensitive to PDMC and PEMC compared to other senescent breast cancer cells. This observation is similar to the sensitivity results of proliferating breast cancer cells treated with 2.5% PDMC and PEMC (Figure 4). As HCC1806 cells are a cellular model of triple negative breast cancer cells with no targeted therapy available [3], encapsulated polyphenolic extract-mediated cytotoxicity against both proliferating and non-proliferating (here doxorubicin-induced senescent) HCC1806 cells seems promising. In contrast to encapsulated polyphenolic extract-treated proliferating breast cancer cells, PDMC and PEMC also promoted a necrotic mode of cell death in senescent HCC1806 and AU565 cells, as judged by 7-AAD staining (Figure 5B). Plant-derived substances (e.g., quercetin, fisetin, piperlongumine, and their derivatives) have been already reported to act against senescent cells and classified as a group of senolytics, agents that can selectively eliminate senescent cells by apoptosis induction [37,38]. More recently, we also showed that quercetin derivative with blocked hydroxy groups potentiated apoptotic cell death in etoposide-induced senescent TNBC cells compared to quercetin treatment [39]. To the best of our knowledge, there are no data on apigenin or luteolin, two of the most abundant flavonoid components of encapsulated polyphenolic extract (Figure S1), mediating senolytic activity in biological systems. However, in our experimental system, we have documented that encapsulated polyphenolic extract can be active against both proliferating (apoptosis induction) and non-proliferating (doxorubicin-induced senescent, apoptosis and/or necrosis induction) breast cancer cells (Figure 4 and Figure 5B).

The effect of empty microcapsules (MC) was also observed in senescent AU565 cells and was similar to results obtained for MC-treated non-senescent AU565 cells (Figure 4). The effects of encapsulated polyphenolic extract were also masked in senescent HCC1428 cells, as MC-mediated apoptosis was also noticed. One can conclude that senescent cells are more sensitive to the treatment with empty microcapsules than corresponding proliferating (non-senescent) breast cancer cells (Figure 4 and Figure 5B). We have also evaluated the levels of two other apoptotic markers, namely the levels of caspase 9 (initiator caspase in mitochondrial pathway of apoptosis) and caspase 3 (executioner caspase both in intrinsic and extrinsic pathways of apoptosis) (Figure 5C). The levels of caspase 9 were not affected in encapsulated polyphenolic extract-treated senescent HCC1806 and HCC1428 cells (Figure 5C and Figure S4). Thus, perhaps, in these cells, encapsulated polyphenolic extract-mediated apoptotic cell death did not involve the mitochondrial pathway. In contrast, in encapsulated polyphenolic extract-treated AU565 cells, a slight increase in the levels of caspase 9 was observed upon PEMC stimulation (Figure 5C). However, a similar result was noticed after MC treatment, which may explain MC-induced cytotoxicity in these cells (Figure 5B,C). An increase in the levels of caspase 3 was observed in PDMC and PEMC-treated AU565 cells and PEMC-treated HCC1428 cells (Figure 5C). Perhaps, in other encapsulated polyphenolic extract-treated cells, apoptosis is mediated by caspases other than caspase 3 executioner caspases (Figure 5C).

As we considered the effects of encapsulated polyphenolic extract in non-proliferating breast cancer cells, the levels of cell cycle inhibitor p21 were also examined after treatment with PDMC and PEMC (Figure 5C). An increase in the levels of nuclear p21 in encapsulated polyphenolic extract-treated HCC1806 cells and PEMC-treated AU565 cells (Figure 5C,D) was accompanied by the most pronounced pro-apoptotic effects in these cells compared to other cells with unaffected levels of p21, for example, encapsulated polyphenolic extract-treated HCC1428 cells (Figure 5B). However, the role of p21 in mediating encapsulated polyphenolic extract-associated cytotoxic effects requires further elucidation, as p21 might be involved in the regulation of cell proliferation, cellular senescence, as well as cell death, depending on cellular context. One can also suggest that encapsulated polyphenolic extract-mediated changes in p21 pools may be associated with the action of apigenin and luteolin-rich pearl millet extracts (Figure S1). Indeed, apigenin and luteolin were found to promote the expression of forkhead box O3 (FOXO3a) and FOXO3a target genes, such as the cyclin-dependent kinase inhibitors p21^Cip1^ (p21) and p27^kip1^ (p27) in breast cancer cells with different receptor status [40]. Apigenin and luteolin-induced expression of p21 was accompanied by an increase in apoptotic markers and related cytotoxicity in breast cancer cells [40].

As encapsulated polyphenolic extract-treated senescent HCC1806 and AU565 cells were also characterized by increased markers of necrotic cell death (7-AAD staining, Figure 5B) that may have immunogenic effects, the levels of proinflammatory cytokine interleukin 8 (IL8) were then assayed (Figure 5C). Indeed, PDMC and PEMC treatment resulted in elevated levels of IL8 in HCC1806 and AU565 cells (Figure 5C). However, increased levels of IL8 were also noted in PEMC-treated HCC1428 cells where necrosis was not revealed. Perhaps the IL8-based response during the treatment with encapsulated polyphenolic extract is not limited to encapsulated polyphenolic extract-mediated necrosis in breast cancer cells. MC treatment was also accompanied by increased levels of IL8 in HCC1806 and AU565 cells (Figure 5C). These results may be considered as a side effect of the use of the microcapsule-based drug delivery system, as increased levels of IL8 may potentiate a senescence-associated secretory phenotype (SASP), a hallmark of cellular senescence [35,41]. These results are surprising, as one can expect that apigenin-rich pearl millet extract would have an anti-inflammatory effect rather than pro-inflammatory activity (Figure 5C). In general, apigenin is considered as an anti-inflammatory agent [32]. Furthermore, apigenin was reported to suppress SASP in stress-induced and replicatively senescent fibroblasts by attenuating L-1α signaling through IRAK1 and IRAK4, p38-MAPK, and NF-κB [42]. Thus, apigenin was classified as a novel senomorphic (senostatic) agent, decreasing pro-inflammatory profiles of senescent cells. Apigenin also limited the ability of SASP to induce cancer cell aggressiveness, namely reduced SASP-mediated paracrine effects on breast cancer cells as judged by decreased aggressiveness of breast cancer cells (e.g., diminished cell proliferation, extracellular matrix invasion, and epithelial–mesenchymal transition) [42]. As a complex IL8-based immune response was observed after the treatment with encapsulated polyphenolic extract (Figure 5C), more studies are needed to characterize pro-inflammatory components of encapsulated polyphenolic extract and related mechanisms.

### 3.5. Encapsulated Polyphenolic Extract-Induced Cytotoxic Autophagy in Senescent AU565 Breast Cancer Cells

**Figure 6:**
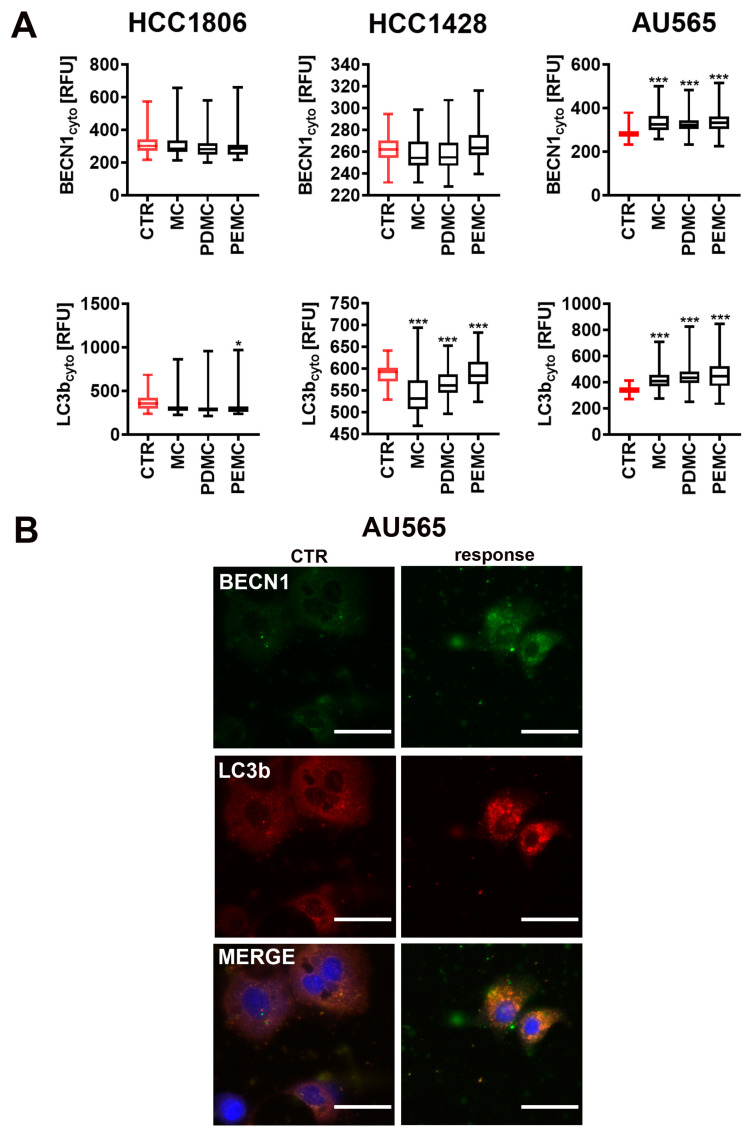
PDMC- and PEMC-mediated changes in the levels of autophagic markers beclin-1 (BECN1) and LC3b in doxorubicin-induced senescent breast cancer cells HCC1806, HCC1428, and AU565. (**A**,**B**) Senescent breast cancer cells were treated with 0.62% microcapsules for 24 h. The levels of cytoplasmic BECN1 and LC3b were analyzed using dedicated antibodies and imaging cytometry. (**A**) Data are presented as relative fluorescence units (RFU). Box and whisker plots are presented, *n* = 6, *** *p* < 0.001, * *p* < 0.05 compared to CTR (ANOVA and Dunnett’s a posteriori test). (**B**) Representative microphotographs of non-treated (CTR) and treated AU565 cells (response) are also shown. BECN1 and LC3b immunosignals are presented in green and red, respectively (immunostaining with dedicated primary antibodies and fluorochrome conjugated secondary antibodies). Nuclei are presented in blue (Hoechst 33342 staining). Objective 20×. CTR, control conditions; MC, treatment with empty microcapsules; PDMC, treatment with encapsulated pearl millet polyphenolic extract dissolved in DMSO; PEMC, treatment with encapsulated pearl millet polyphenolic extract dissolved in EtOH.

As autophagy may exert both beneficial (e.g., cytotoxic autophagy) and detrimental (e.g., protective autophagy) effects during chemotherapeutic treatment and there are interconnections between apoptosis, autophagy, and senescence [43,44], we also investigated the levels of two autophagic markers, namely beclin-1 and LC3b, in encapsulated polyphenolic extract-treated senescent breast cancer cells (Figure 6). We found that senescent breast cancer cells responded differently to the treatment with encapsulated polyphenolic extract in terms of autophagy induction (Figure 6). In encapsulated polyphenolic extract-treated senescent AU565 cells, the autophagic pathway was activated, as judged by increased levels of beclin-1 and LC3b (Figure 6A,B). This autophagic response may be considered as a cytotoxic autophagy, as beclin-1- and LC3b-mediated autophagy was accompanied by apoptotic cell death in encapsulated polyphenolic extract-treated senescent AU565 cells (Figure 5B and Figure 6). A similar effect was not observed in encapsulated polyphenolic extract-treated senescent HCC1806 and HCC1428 cells (Figure 6A and Figure S5). Perhaps, in these cells, encapsulated polyphenolic extract-mediated cytotoxicity is not associated with the induction of autophagic pathway. There are no data on pearl millet extract or isolated component-mediated autophagy. More recently, bound polyphenols from the inner shell of foxtail millet bran (BPIS), namely ferulic acid (FA) and *p*-coumaric acid (*p*-CA), were reported to induce autophagy-mediated cytotoxicity in breast cancer cells [45]. Mechanistically, BPIS-associated FA and *p*-CA stimulated the expression of choline-phosphate cytidylyltransferase A (PCYT1A), a key enzyme of glycerophospholipid synthesis, leading to lipid accumulation, extensive autophagy (in this case, lipophagy, an autophagy-associated degradation of lipids), and autophagic death of breast cancer MCF-7 and MDA-MB-231 cells [45]. The leaf extract of the European black nightshade (*Solanum nigrum* L.), rich in gentisic acid, luteolin, apigenin, kaempferol, and *m*-coumaric acid (>100 µg/mL), also exerted cytotoxic effects against AU565 cells by the induction of both autophagy and apoptosis [46]. Perhaps apigenin present in high concentrations in pearl millet extract (Figure S1) may also contribute to encapsulated polyphenolic extract-mediated cytotoxic autophagy in AU565 cells (this study). Indeed, apigenin was shown to promote autophagy in a number of cancer cell types [32]. However, the therapeutic outcomes were dependent on cellular context [32]. For example, apigenin also induced autophagy and apoptosis in TNBC MDA-MB-231 cells, but autophagy inhibition by 3-methyladenine treatment potentiated apigenin-induced apoptosis in these cells [32]. This result may suggest that apigenin promoted an adaptive and cytoprotective response in MDA-MB-231 cells.

More studies are needed to reveal the molecular mechanisms underlying encapsulated polyphenolic extract-mediated anticancer action, especially the involvement of cell death-associated signaling pathways.

## 4. Conclusions

**Figure 7:**
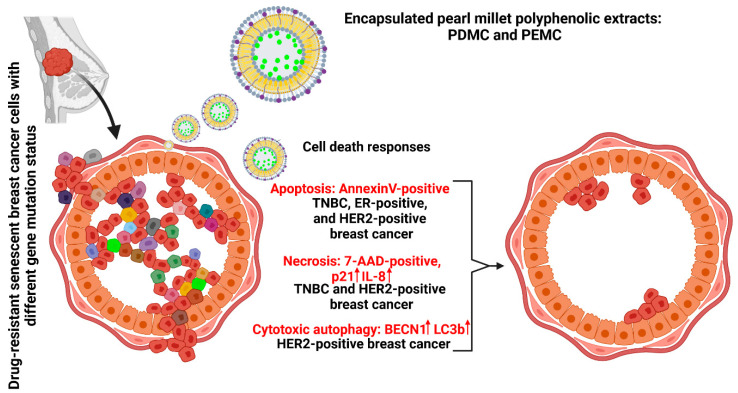
Diverse cell death responses in drug-induced senescent breast cancer cells after treatment with encapsulated pearl millet polyphenolic extracts (PDMC and PEMC). Three phenotypically different cellular models of breast cancer were considered, namely TNBC (HCC1806 cells), ER-positive (HCC1428 cells), and HER2-positive (AU565 cells). Encapsulated polyphenolic extract induced apoptosis in all three breast cancer cell lines. Necrosis was also observed in encapsulated polyphenolic extract-treated TNBC and HER2-positive cells, which was accompanied by increased levels of p21 and IL8. Encapsulated polyphenolic extract-induced decrease in cell viability was also accompanied by the activation of autophagic pathway, here cytotoxic autophagy, in HER2-positive cells. Encapsulated polyphenolic extract also promoted apoptotic cell death in proliferating breast cancer cells and pro-apoptotic activity of encapsulated polyphenolic extract was potentiated compared to the cytotoxic effects induced by free polyphenolic fractions. PDMC, treatment with encapsulated pearl millet polyphenolic extract dissolved in DMSO; PEMC, treatment with encapsulated pearl millet polyphenolic extract dissolved in EtOH.

We have obtained and characterized the polyphenol-rich fraction (apigenin, caffeic acid, and luteolin) from the pearl millet grains and tested its anticancer activity against phenotypically different breast cancer cells (triple negative HCC1806, ER-positive HCC1428, and HER2-positive AU565) using a microcapsule-based drug delivery system in vitro. Two encapsulated polyphenolic extracts dissolved in DMSO and EtOH were considered. The microencapsulation approach potentiated the anticancer effects of pearl millet polyphenolic extracts. Pro-apoptotic activity of PDMC and PEMC was revealed in both proliferating and drug-resistant senescent breast cancer cells (Figure 7). The anticancer effects of encapsulated polyphenolic extract were accompanied by increased levels of p21 and IL8, and, in the case of AU565 cells, the induction of cytotoxic autophagy (Figure 7). As the treatment with encapsulated polyphenolic extract was the most effective against triple negative HCC1806 cells and ineffective against non-tumorigenic MCF10F cells, we postulate that encapsulated polyphenolic extract may be considered in the future as a part of a novel strategy against triple negative breast cancer with limited therapeutic targeting options. More studies are needed to validate such assumptions.

## Data Availability

The raw data supporting the conclusions of this article will be made available by the authors on request.

## Supplementary Materials

The following supporting information can be downloaded at: https://www.mdpi.com/article/10.3390/cancers16091750/s1, Figure S1. HPLC chromatograms of pearl millet polyphenol extracts dissolved in ethanol. Figure S2. Size distribution of microcapsules MC, PDMC, and PEMC. Figure S3. Confirmation of the activation of drug-induced senescence program in three breast cancer cell lines using senescence-associated beta-galactosidase (SA-beta-gal) assay and imaging flow cytometry. Figure S4. Representative microphotographs of non-treated (CTR) and treated HCC1806 and HCC1428 cells (response) of Casp9, Casp3, p21, and IL8 immunostaining. Figure S5. Representative microphotographs of non-treated (CTR) and treated HCC1806 and HCC1428 cells (response) of BECN1 and LC3b immunostaining. Table S1. Raman vibrational frequencies of the studied samples.

## Abbreviations

ANOVA—one-way analysis of variance, BPIS—bound polyphenols from the inner shell of foxtail millet bran, DMSO—dimethyl sulfoxide, DW—dry weight, ER—estrogen receptor, FA—ferulic acid, HER2—human epidermal growth factor receptor 2, MC—empty microcapsules, *p*-CA—*p*-coumaric acid, PCYT1A—choline-phosphate cytidylyltransferase A, PD—free pearl millet extracts dissolved in DMSO, PDMC—encapsulated pearl millet polyphenolic extract dissolved in DMSO, PE—free pearl millet extracts dissolved in ethanol, PEMC—encapsulated pearl millet polyphenolic extract dissolved in EtOH, PL—free pearl millet extracts dissolved in lipids, PM—pearl millet, RFU—relative fluorescence units, ROS -reactive oxygen species, SASP—senescence-associated secretory phenotype, TIS—therapy-induced senescence, TNBC—triple negative breast cancer, TSA—tryptone soya agar.

## References

[1] Siegel R.L., Giaquinto A.N., Jemal A. (2024). Cancer Statistics, 2024. CA. Cancer J. Clin..

[2] Yardim-Akaydin S., Karahalil B., Baytas S.N. (2022). New Therapy Strategies in the Management of Breast Cancer. Drug Discov. Today.

[3] Dai X., Cheng H., Bai Z., Li J. (2017). Breast Cancer Cell Line Classification and Its Relevance with Breast Tumor Subtyping. J. Cancer.

[4] Yin L., Duan J.-J., Bian X.-W., Yu S. (2020). Triple-Negative Breast Cancer Molecular Subtyping and Treatment Progress. Breast Cancer Res..

[5] Bianchini G., De Angelis C., Licata L., Gianni L. (2022). Treatment Landscape of Triple-Negative Breast Cancer—Expanded Options, Evolving Needs. Nat. Rev. Clin. Oncol..

[6] Choudhari A.S., Mandave P.C., Deshpande M., Ranjekar P., Prakash O. (2020). Phytochemicals in Cancer Treatment: From Preclinical Studies to Clinical Practice. Front. Pharmacol..

[7] Maleki Dana P., Sadoughi F., Asemi Z., Yousefi B. (2022). The Role of Polyphenols in Overcoming Cancer Drug Resistance: A Comprehensive Review. Cell. Mol. Biol. Lett..

[8] Cháirez-Ramírez M.H., de la Cruz-López K.G., García-Carrancá A. (2021). Polyphenols as Antitumor Agents Targeting Key Players in Cancer-Driving Signaling Pathways. Front. Pharmacol..

[9] Farghadani R., Naidu R. (2023). The Anticancer Mechanism of Action of Selected Polyphenols in Triple-Negative Breast Cancer (TNBC). Biomed. Pharmacother..

[10] Estrela J.M., Mena S., Obrador E., Benlloch M., Castellano G., Salvador R., Dellinger R.W. (2017). Polyphenolic Phytochemicals in Cancer Prevention and Therapy: Bioavailability versus Bioefficacy. J. Med. Chem..

[11] Wu X., Li M., Xiao Z., Daglia M., Dragan S., Delmas D., Vong C.T., Wang Y., Zhao Y., Shen J. (2020). Dietary Polyphenols for Managing Cancers: What Have We Ignored?. Trends Food Sci. Technol..

[12] Faridi Esfanjani A., Jafari S.M. (2016). Biopolymer Nano-Particles and Natural Nano-Carriers for Nano-Encapsulation of Phenolic Compounds. Colloids Surf. B Biointerfaces.

[13] Rezaei A., Fathi M., Jafari S.M. (2019). Nanoencapsulation of Hydrophobic and Low-Soluble Food Bioactive Compounds within Different Nanocarriers. Food Hydrocoll..

[14] Khan H., Ullah H., Martorell M., Valdes S.E., Belwal T., Tejada S., Sureda A., Kamal M.A. (2021). Flavonoids Nanoparticles in Cancer: Treatment, Prevention and Clinical Prospects. Semin. Cancer Biol..

[15] Pimentel-Moral S., Teixeira M.C., Fernandes A.R., Arráez-Román D., Martínez-Férez A., Segura-Carretero A., Souto E.B. (2018). Lipid Nanocarriers for the Loading of Polyphenols—A Comprehensive Review. Adv. Colloid Interface Sci..

[16] Puligundla P., Mok C., Ko S., Liang J., Recharla N. (2017). Nanotechnological Approaches to Enhance the Bioavailability and Therapeutic Efficacy of Green Tea Polyphenols. J. Funct. Foods.

[17] Shahidi F., Chandrasekara A. (2013). Millet Grain Phenolics and Their Role in Disease Risk Reduction and Health Promotion: A Review. J. Funct. Foods.

[18] Nithiyanantham S., Kalaiselvi P., Mahomoodally M.F., Zengin G., Abirami A., Srinivasan G. (2019). Nutritional and Functional Roles of Millets—A Review. J. Food Biochem..

[19] Majid A., Priyadarshini C.G.P. (2020). Millet Derived Bioactive Peptides: A Review on Their Functional Properties and Health Benefits. Crit. Rev. Food Sci. Nutr..

[20] Samtiya M., Aluko R.E., Dhaka N., Dhewa T., Puniya A.K. (2023). Nutritional and Health-Promoting Attributes of Millet: Current and Future Perspectives. Nutr. Rev..

[21] Nani A., Belarbi M., Ksouri-Megdiche W., Abdoul-Azize S., Benammar C., Ghiringhelli F., Hichami A., Khan N.A. (2015). Effects of Polyphenols and Lipids from *Pennisetum Glaucum* Grains on T-Cell Activation: Modulation of Ca^2+^ and ERK1/ERK2 Signaling. BMC Complement. Altern. Med..

[22] Aires A., Carvalho R. (2020). Kiwi Fruit Residues from Industry Processing: Study for a Maximum Phenolic Recovery Yield. J. Food Sci. Technol..

[23] Hudecki A., Rzeszutek I., Lewińska A., Warski T., Baranowska-Korczyc A., Wojnarowska-Nowak R., Betlej G., Deręgowska A., Hudecki J., Łyko-Morawska D. (2023). Electrospun Fiber-Based Micro- and Nano-System for Delivery of High Concentrated Quercetin to Cancer Cells. Biomater. Adv..

[24] Przybylski P., Lewińska A., Rzeszutek I., Błoniarz D., Moskal A., Betlej G., Deręgowska A., Cybularczyk-Cecotka M., Szmatoła T., Litwinienko G. (2023). Mutation Status and Glucose Availability Affect the Response to Mitochondria-Targeted Quercetin Derivative in Breast Cancer Cells. Cancers.

[25] Kuete V., Tchinda C.F., Mambe F.T., Beng V.P., Efferth T. (2016). Cytotoxicity of Methanol Extracts of 10 Cameroonian Medicinal Plants towards Multi-Factorial Drug-Resistant Cancer Cell Lines. BMC Complement. Altern. Med..

[26] Shi J., Shan S., Li Z., Li H., Li X., Li Z. (2015). Bound Polyphenol from Foxtail Millet Bran Induces Apoptosis in HCT-116 Cell through ROS Generation. J. Funct. Foods.

[27] Espina A., Sanchez-Cortes S., Jurašeková Z. (2022). Vibrational Study (Raman, SERS, and IR) of Plant Gallnut Polyphenols Related to the Fabrication of Iron Gall Inks. Molecules.

[28] Corredor C., Teslova T., Cañamares M.V., Chen Z., Zhang J., Lombardi J.R., Leona M. (2009). Raman and Surface-Enhanced Raman Spectra of Chrysin, Apigenin and Luteolin. Vib. Spectrosc..

[29] de Siqueira e Oliveira F.S., Giana H.E., Silveira L. (2012). Discrimination of Selected Species of Pathogenic Bacteria Using Near-Infrared Raman Spectroscopy and Principal Components Analysis. J. Biomed. Opt..

[30] Schönemann A., Edwards H.G.M. (2011). Raman and FTIR Microspectroscopic Study of the Alteration of Chinese Tung Oil and Related Drying Oils during Ageing. Anal. Bioanal. Chem..

[31] Siwak J., Lewinska A., Wnuk M., Bartosz G. (2013). Protection of Flavonoids against Hypochlorite-Induced Protein Modifications. Food Chem..

[32] Ahmed S.A., Parama D., Daimari E., Girisa S., Banik K., Harsha C., Dutta U., Kunnumakkara A.B. (2021). Rationalizing the Therapeutic Potential of Apigenin against Cancer. Life Sci..

[33] Hong S., Dia V.P., Baek S.J., Zhong Q. (2022). Nanoencapsulation of Apigenin with Whey Protein Isolate: Physicochemical Properties, in Vitro Activity against Colorectal Cancer Cells, and Bioavailability. LWT.

[34] Adel M., Zahmatkeshan M., Akbarzadeh A., Rabiee N., Ahmadi S., Keyhanvar P., Rezayat S.M., Seifalian A.M. (2022). Chemotherapeutic Effects of Apigenin in Breast Cancer: Preclinical Evidence and Molecular Mechanisms; Enhanced Bioavailability by Nanoparticles. Biotechnol. Rep..

[35] Wang B., Kohli J., Demaria M. (2020). Senescent Cells in Cancer Therapy: Friends or Foes?. Trends Cancer.

[36] Wang L., Lankhorst L., Bernards R. (2022). Exploiting Senescence for the Treatment of Cancer. Nat. Rev. Cancer.

[37] Zhu Y., Tchkonia T., Pirtskhalava T., Gower A.C., Ding H., Giorgadze N., Palmer A.K., Ikeno Y., Hubbard G.B., Lenburg M. (2015). The Achilles’ Heel of Senescent Cells: From Transcriptome to Senolytic Drugs. Aging Cell.

[38] Zhang L., Pitcher L.E., Prahalad V., Niedernhofer L.J., Robbins P.D. (2021). Recent Advances in the Discovery of Senolytics. Mech. Ageing Dev..

[39] Lewińska A., Przybylski P., Adamczyk-Grochala J., Błoniarz D., Litwinienko G., Wnuk M. (2022). Senolysis-Based Elimination of Chemotherapy-Induced Senescent Breast Cancer Cells by Quercetin Derivative with Blocked Hydroxy Groups. Cancers.

[40] Lin C.-H., Chang C.-Y., Lee K.-R., Lin H.-J., Chen T.-H., Wan L. (2015). Flavones Inhibit Breast Cancer Proliferation through the Akt/FOXO3a Signaling Pathway. BMC Cancer.

[41] Coppé J.-P., Patil C.K., Rodier F., Sun Y., Muñoz D.P., Goldstein J., Nelson P.S., Desprez P.-Y., Campisi J. (2008). Senescence-Associated Secretory Phenotypes Reveal Cell-Nonautonomous Functions of Oncogenic RAS and the P53 Tumor Suppressor. PLoS Biol..

[42] Perrott K.M., Wiley C.D., Desprez P.-Y., Campisi J. (2017). Apigenin Suppresses the Senescence-Associated Secretory Phenotype and Paracrine Effects on Breast Cancer Cells. GeroScience.

[43] Gewirtz D.A. (2014). The Four Faces of Autophagy: Implications for Cancer Therapy. Cancer Res..

[44] Vicencio J.M., Galluzzi L., Tajeddine N., Ortiz C., Criollo A., Tasdemir E., Morselli E., Ben Younes A., Maiuri M.C., Lavandero S. (2008). Senescence, Apoptosis or Autophagy?. Gerontology.

[45] Zhang L., Liu Y., La X., Li S., Wen L., Liu T., Li H., Li A., Wu H., Wu C. (2023). The Bound Polyphenols of Foxtail Millet (*Setaria Italica*) Inner Shell Inhibit Breast Cancer by Promoting Lipid Accumulation-Induced Autophagic Death. Food Chem. Toxicol..

[46] Huang H.-C., Syu K.-Y., Lin J.-K. (2010). Chemical Composition of *Solanum Nigrum* Linn Extract and Induction of Autophagy by Leaf Water Extract and Its Major Flavonoids in AU565 Breast Cancer Cells. J. Agric. Food Chem..

