# Supplementary material for: Anticancer Activity of Encapsulated Pearl Millet Polyphenol-Rich Extract against Proliferating and Non-Proliferating Breast Cancer Cells In Vitro
Source: Cancers (Basel). 2024 Apr 30;16(9):1750. doi: 10.3390/cancers16091750 (PMC11083001; doi:10.3390/cancers16091750)
Supplement: Supplementary file 1 [file cancers-16-01750-s001.zip › cancers-2973627-supplementary.pdf]

Supplementary materials

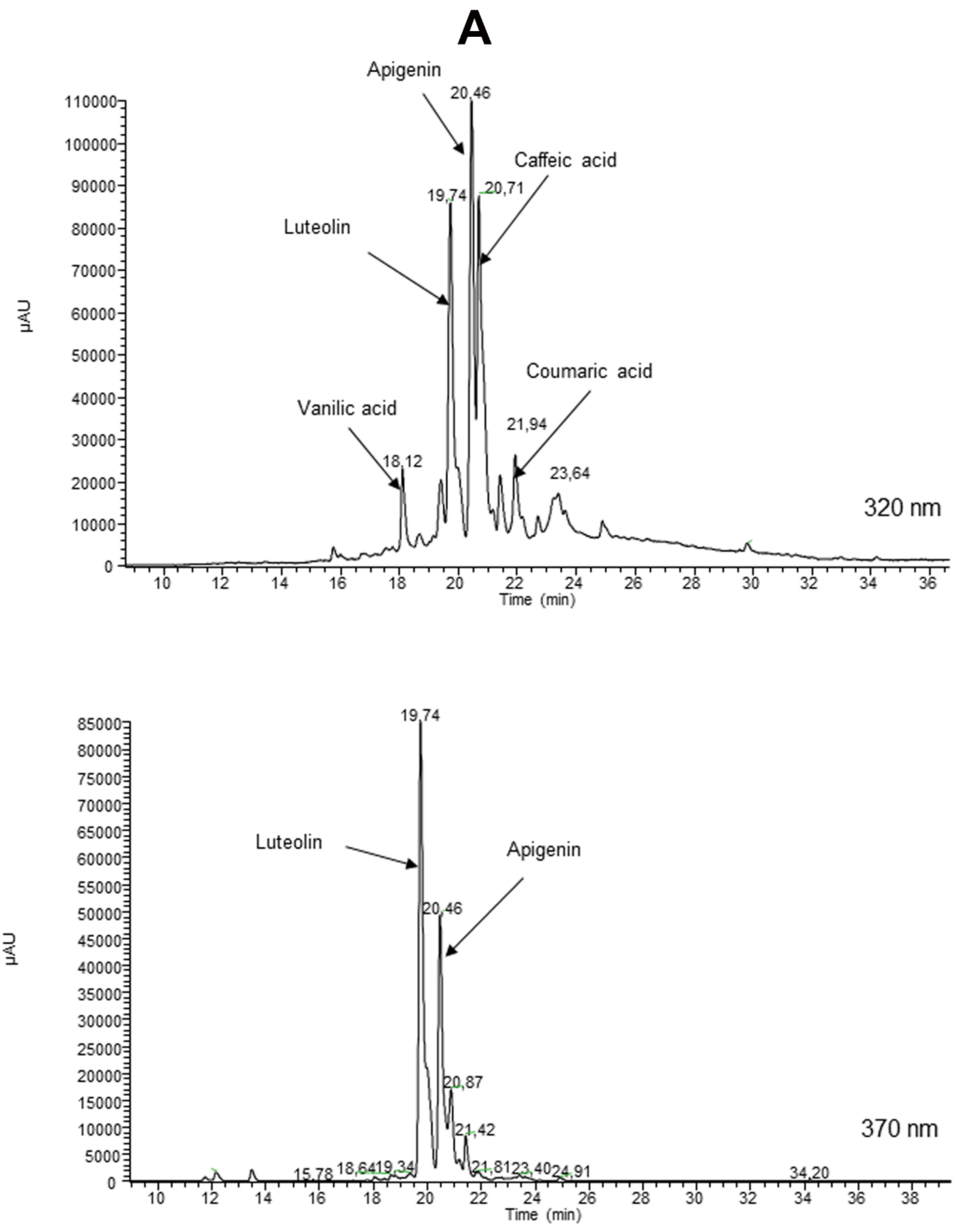

Soluble phenolics

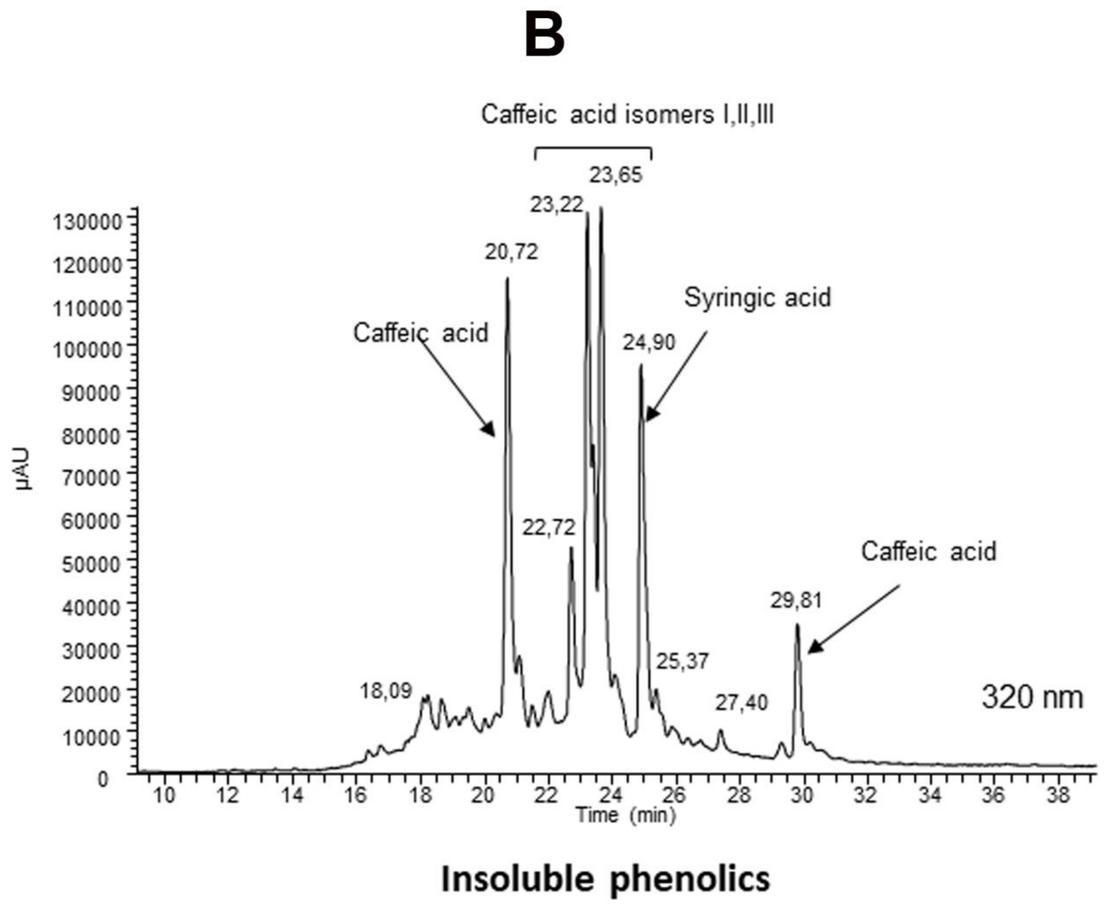

Supplementary Figure S1. HPLC chromatograms of pearl millet polyphenol extracts dissolved in ethanol, measured at different wavelengths (320 nm for cinnamic acids and 370 nm for flavonoids). The fractions of soluble (A) and insoluble (B) phenolics are shown.

A

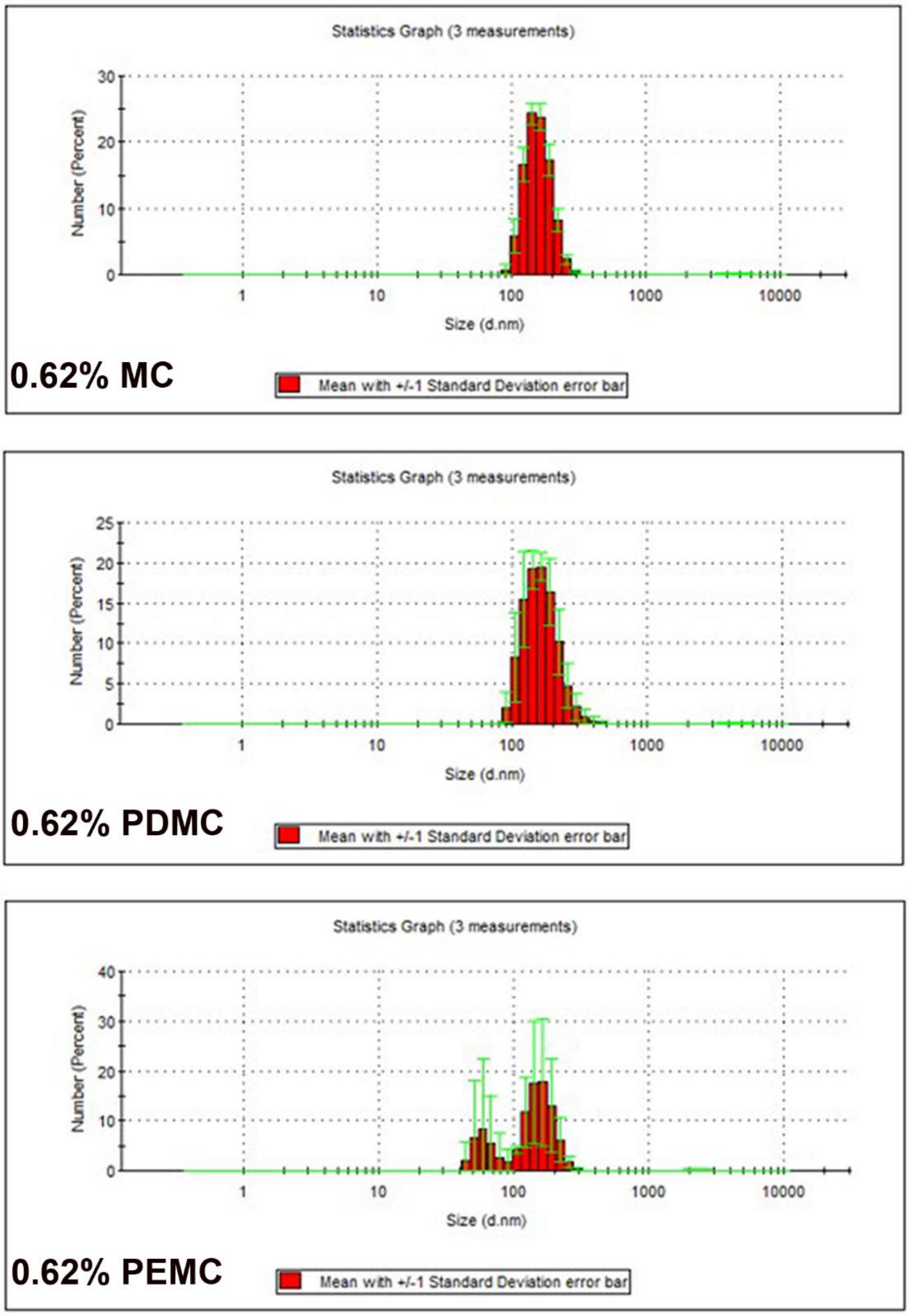
Statistics Graph (3 measurements)
Number (Percent)
Size (d.nm)
0.62% MC
Mean with +/-1 Standard Deviation error bar
Statistics Graph (3 measurements)
Number (Percent)
Size (d.nm)
0.62% PDMC
Mean with +/-1 Standard Deviation error bar
Statistics Graph (3 measurements)
Number (Percent)
Size (d.nm)
0.62% PEMC
Mean with +/-1 Standard Deviation error bar

B

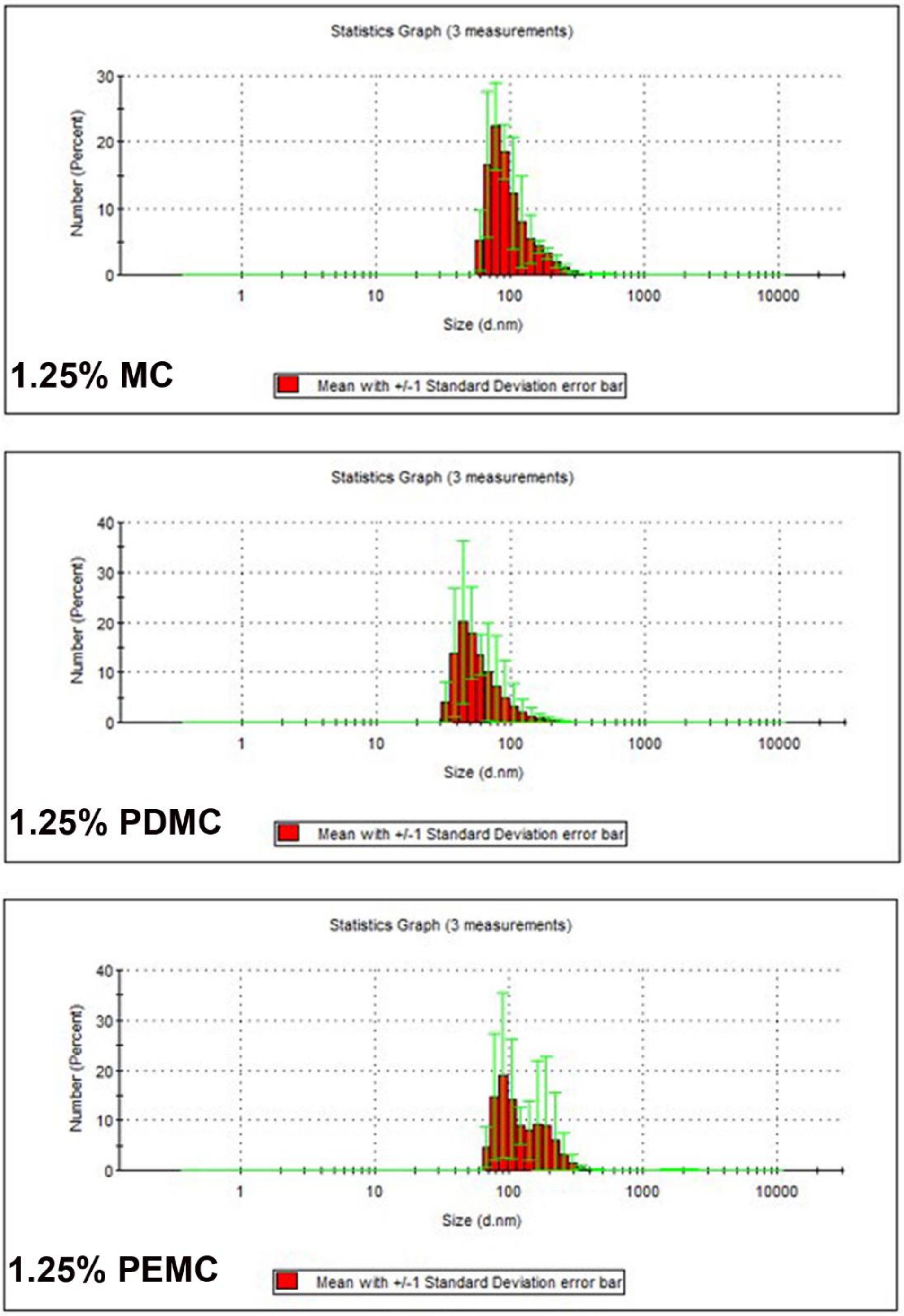
Statistics Graph (3 measurements)
Number (Percent)
30
20
10
0
1
10
100
1000
10000
Size (d.nm)
1.25% MC
Mean with +/-1 Standard Deviation error bar
Statistics Graph (3 measurements)
Number (Percent)
40
30
20
10
0
1
10
100
1000
10000
Size (d.nm)
1.25% PDMC
Mean with +/-1 Standard Deviation error bar
Statistics Graph (3 measurements)
Number (Percent)
40
30
20
10
0
1
10
100
1000
10000
Size (d.nm)
1.25% PEMC
Mean with +/-1 Standard Deviation error bar

C

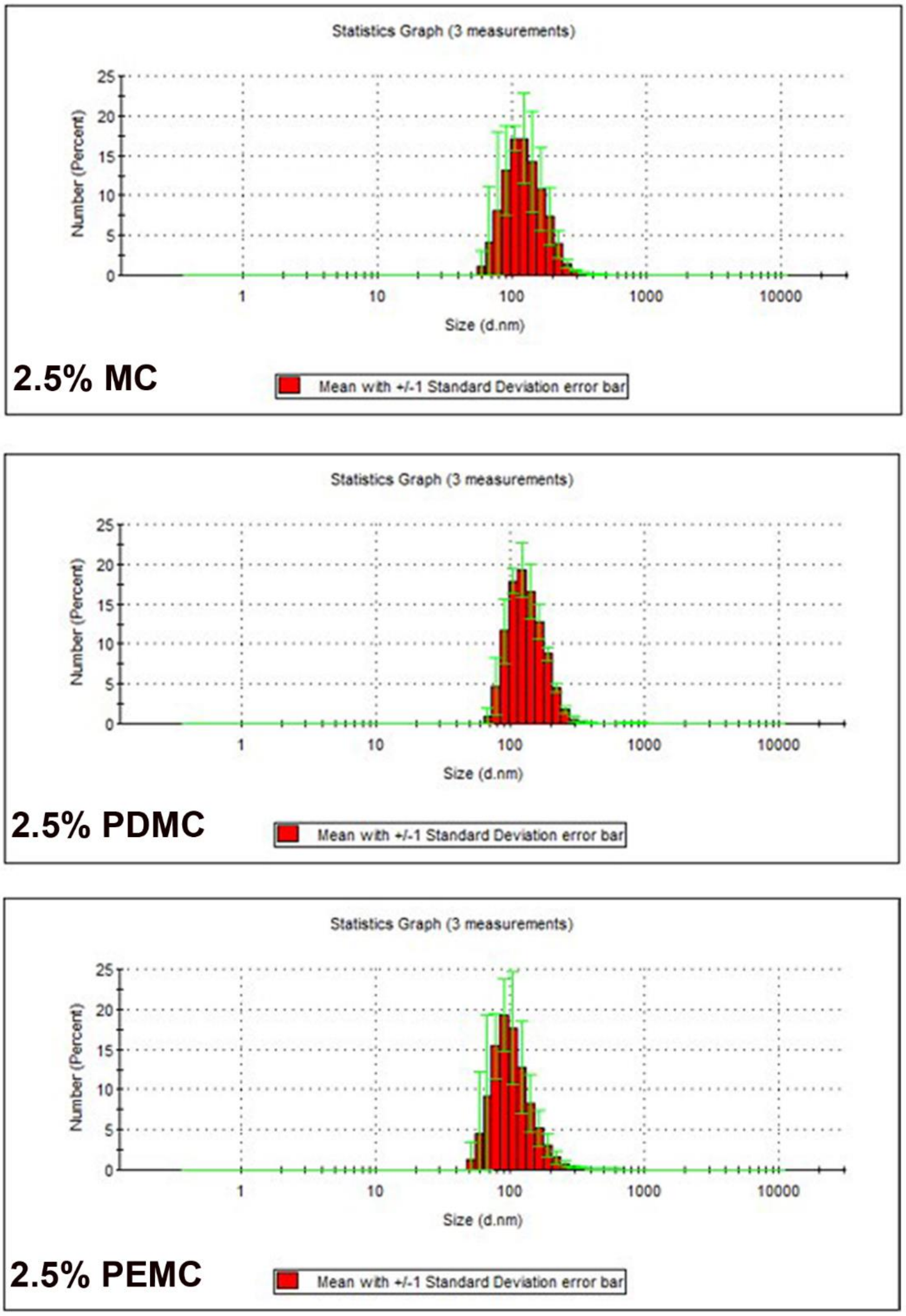

Supplementary Figure S2. Size distribution of microcapsules MC, PDMC, and PEMC at the concentrations of 0.62% (A), 1.25% (B), and 2.5% (C). Size distribution of microcapsules was analyzed using dynamic light scattering (DLS). Microcapsule size is expressed as a hydrodynamic diameter.

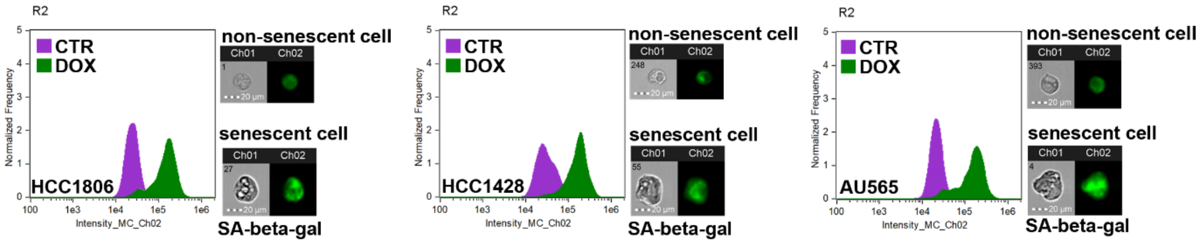

Supplementary Figure S3. Confirmation of the activation of drug-induced senescence program in three breast cancer cell lines using senescence-associated beta-galactosidase (SA-beta-gal) assay and imaging flow cytometry. Representative histograms and microphotographs are shown. CTR, control conditions; DOX, treatment with doxorubicin.

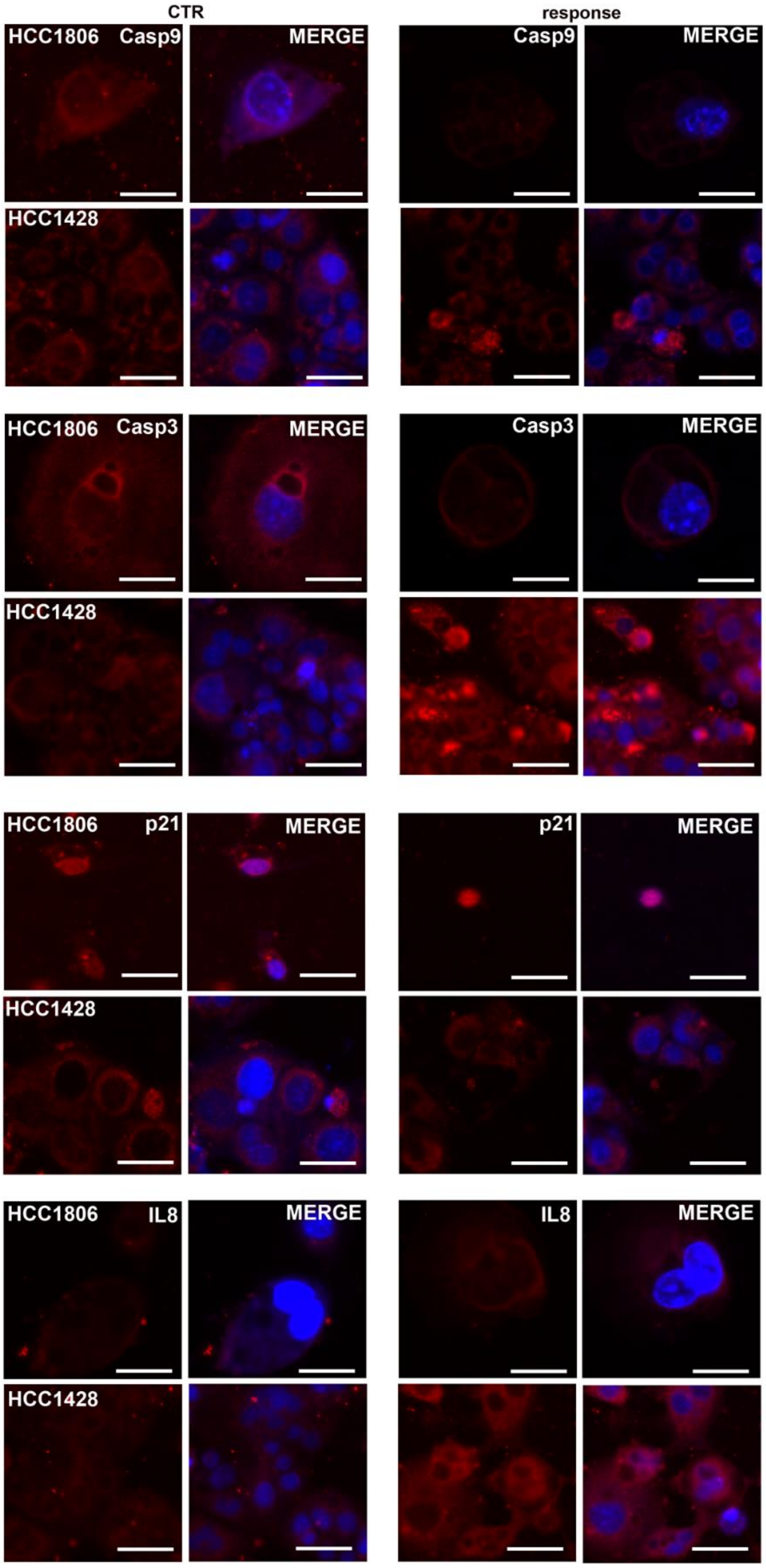

Supplementary Figure S4. Representative microphotographs of non-treated (CTR) and treated HCC1806 and HCC1428 cells (response) of Casp9, Casp3, p21, and IL8 immunostaining. Immunosignals are presented in red (immunostaining with dedicated primary antibodies and fluorochrome conjugated secondary antibodies). Nuclei are presented in blue (Hoechst 33342 staining). Objective 20×.

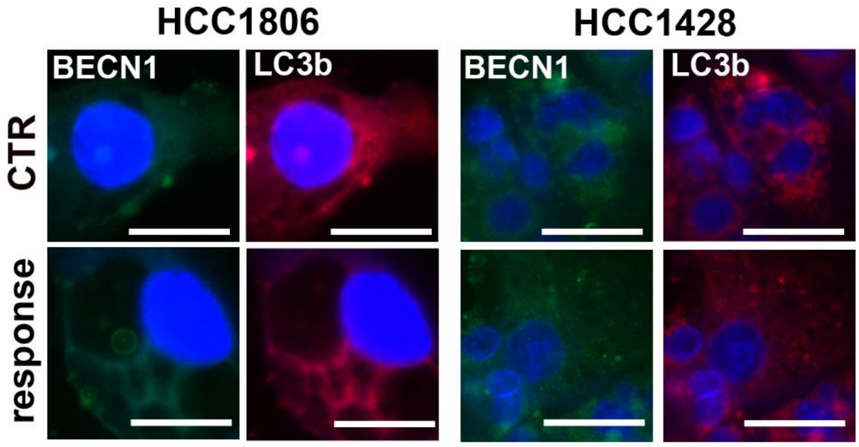

Supplementary Figure S5. Representative microphotographs of non-treated (CTR) and treated HCC1806 and HCC1428 cells (response) of BECN1 and LC3b immunostaining. Immunosignals are presented in green and red, respectively (immunostaining with dedicated primary antibodies and fluorochrome conjugated secondary anti-bodies). Nuclei are presented in blue (Hoechst 33342 staining). Objective 20×.

Supplementary Table S1. Raman vibrational frequencies of the studied samples (MC, MC*, PDMC*, PEMC*).

**Raman shift (cm^{-1})**				**Vibrational Assignment**	**Molecules**
MC	MC*	PDMC*	PEMC*		
-	-	307	309	δ(CCC)	polyphenol
-	366	358	358	ring bending modes	FL
-	406	410	410	ring bending modes	FL
453	453 (sh)	451 (sh)	450 (sh)	-	microcapsule molecules
-	467	464	462	ring bending modes	FL
493	492	495	492	COO, skeletal vibration	microcapsule molecules, polyphenol
604	602	603 (sh)	603 (sh)	-	microcapsule molecules
-	643	642	642	ring bending modes	FL
-	-	677	678	γ(OH)	polyphenol
	-	710	713	ν(ring breathing)	polyphenol
742	742	741	743	CC skeletal deformation, δ (CH_2)	TSA
-	768	765	763	γ (C–H) bending	FL
844	845	845	846	CC deformation/OCO wagging/CH vibrations with C–OH	TSA
867	862 (sh)	864	864 (sh)	-	microcapsule molecules
891	891	991	893	CCH deformation	TSA
-	938	935	938	CH_2 rocking	FL
972	969	969	966	CCH deformation	TSA
1083	1083	1082	1083	ν (C–C)	TSA, linseed oil
-	1171	1173	1172	δ (C–H) bending	FL
1265	1268	1268	1268	ν (C–O) in C–O–C, δ (=C–H)	linseed oil
1305	1302 (sh)	1302 (sh)	1301 (sh)	COH deformation, ν (CCO)	TSA
-	1312	1311	1311	C-C xanthene tension	FL
-	-	1417	1418	ν(ring), δ(OH)	polyphenol
1444	1441	1443	1444	δ (C–H)	TSA, linseed oil
-	1506	1506	1507	ν (C=C) ring	FL
1659	1659	1659	1659	ν (C=C), ν (C–O)	TSA, linseed oil
1747	1747	1746	1747	ν (C=O)	linseed oil
2853	2853	2853	2853	ν (C–H)	TSA, linseed oil
2890	2890	2890	2890	ν (C–H)	TSA, linseed oil
-	-	2922	2920	ν (C–H)	polyphenol

2937	2937 (sh)	2937 (sh)	2937 (sh)	ν (C–H)	TSA, linseed oil
3015	3013	3013	3014 (sh)	ν (C–H) in C=C–H	linseed oil, polyphenol

sh, shoulder; ν, stretching; δ, in-plane bending/deformation; γ, out-of-plane bending; the assignments were made on the basis of references [27-30].
